# A Novel Dual Eigen-Analysis of Mouse Multi-Tissues’ Expression Profiles Unveils New Perspectives into Type 2 Diabetes

**DOI:** 10.1038/s41598-017-05405-x

**Published:** 2017-07-11

**Authors:** Lei M. Li, Xiuxiu Liu, Lin Wang, Yong Wang, Xiuqin Liu, Xue Tian, Fuzhou Gong, Li Shen, Xiao-ding Peng

**Affiliations:** 1 National Center of Mathematics and Interdisciplinary Sciences, Academy of Mathematics and Systems Science, Chinese Academy of Sciences, Beijing, 100190 China; 2 University of Chinese Academy of Sciences, Beijing, 100049 China; 3 Department of Cell Biology, Peking University Health Science Center, Beijing, 100191 China; 4 Department of Otorhinolaryngology Head and Neck Surgery, Shandong Provincial Hospital Affiliated to Shandong University, Shandong Provincial Key Laboratory of Otology, Jinan, 250021 China; 5 School of Statistics, Capital University of Economics and Business, Beijing, China; 6 School of Mathematics and Physics, University of Science and Technology Beijing, Beijing, China; 7 Department of Biochemistry and Molecular Genetics, The University of Illinois at Chicago, Chicago, IL 60607 USA

## Abstract

Type 2 diabetes (T2D) is a complex and polygenic disease yet in need of a complete picture of its development mechanisms. To better understand the mechanisms, we examined gene expression profiles of multi-tissues from outbred mice fed with a high-fat diet (HFD) or regular chow at weeks 1, 9, and 18. To analyze such complex data, we proposed a novel dual eigen-analysis, in which the sample- and gene-eigenvectors correspond respectively to the macro- and micro-biology information. The dual eigen-analysis identified the HFD eigenvectors as well as the endogenous eigenvectors for each tissue. The results imply that HFD influences the hepatic function or the pancreatic development as an exogenous factor, while in adipose HFD’s impact roughly coincides with the endogenous eigenvector driven by aging. The enrichment analysis of the eigenvectors revealed diverse HFD impact on the three tissues over time. The diversity includes: inflammation, degradation of branched chain amino acids (BCAA), and regulation of peroxisome proliferator activated receptor gamma (PPARγ). We reported that in the pancreas remarkable up-regulation of angiogenesis as downstream of the HIF signaling pathway precedes hyperinsulinemia. The dual eigen-analysis and discoveries provide new evaluations/guidance in T2D prevention and therapy, and will also promote new thinking in biology and medicine.

## Introduction

Type 2 diabetes (T2D) is a complex and polygenic disease usually associated with obesity. Its pathological status is characterized by insulin resistance and pancreatic dysfunction, causing the loss of metabolic fuel homeostasis. Various measures have been applied to treat T2D, but as of now none of them can completely cure or reverse this progressive disease^1^. Many efforts have been made to elucidate the mechanisms of insulin resistance and aberrant insulin production/secretion at the molecular level^2, 3^. However, in order to identify early warning signals of this disease along its progression and to provide clues to cure this disease^4, 5^, we need more complete and comprehensive pictures of the systematic and longitudinal impacts of genetic or environmental adverse factors such as chronic overnutrition. It is of significant challenges in designing pharmacological interventions of this disease that some molecular and cellular impacts are more tissue-specific^4^. Also the timing of these impacts is crucial for early diagnosis and treatment. Such systematic picture of the disease progression is almost impossible to draw using only molecular biology approaches. With the help of high throughput technology that can generate big omic data, scientists can study major complex diseases such as T2D using the approaches of computational biology and bioinformatics.

In this study, we focused on the tissue specific characteristics of the gene expression profiles during the development and progresses of T2D among three tissues, two of which are insulin responsive ones, the adipose and the liver; and one of which is the pancreas containing insulin producing part pancreatic islets. We collected data of gene expression profiles obtained from a high fat diet (HFD) induced T2D model generated from an outbred mouse strain which, unlike the knockout approach commonly seen in other literatures, more closely mimics natural human polygenic population^6^, at different time points. We preprocessed the raw microarray data using a statistical method that we developed and tested over the past decade^7–10^ to obtain unbiased and accurate gene expression values. Moreover, we proposed a novel computational methodology – a dual eigen-analysis –in order to extract biological insights from the big data of expression profiles. The usage of computational biology and bioinformatics enabled us for the first time to identify certain diverse impact the HFD had on the three different tissues at different time points. The diversity or tissue specific characteristics includes: inflammation, the degradation of branched chain amino acids (BCAA), and the regulation of peroxisome proliferator activated receptor gamma (PPARγ). Interestingly, we discovered that in the pancreas remarkable up-regulation of angiogenic genes, which are downstream of the HIF (hypoxia-inducible factor) signaling pathway, precedes hyperinsulinemia. These findings may provide new guidance in evaluating and designing pharmacological interventions for T2D prevention and therapy.

## Results

### Tissue-specific expression data of mouse samples

**Figure 1:**
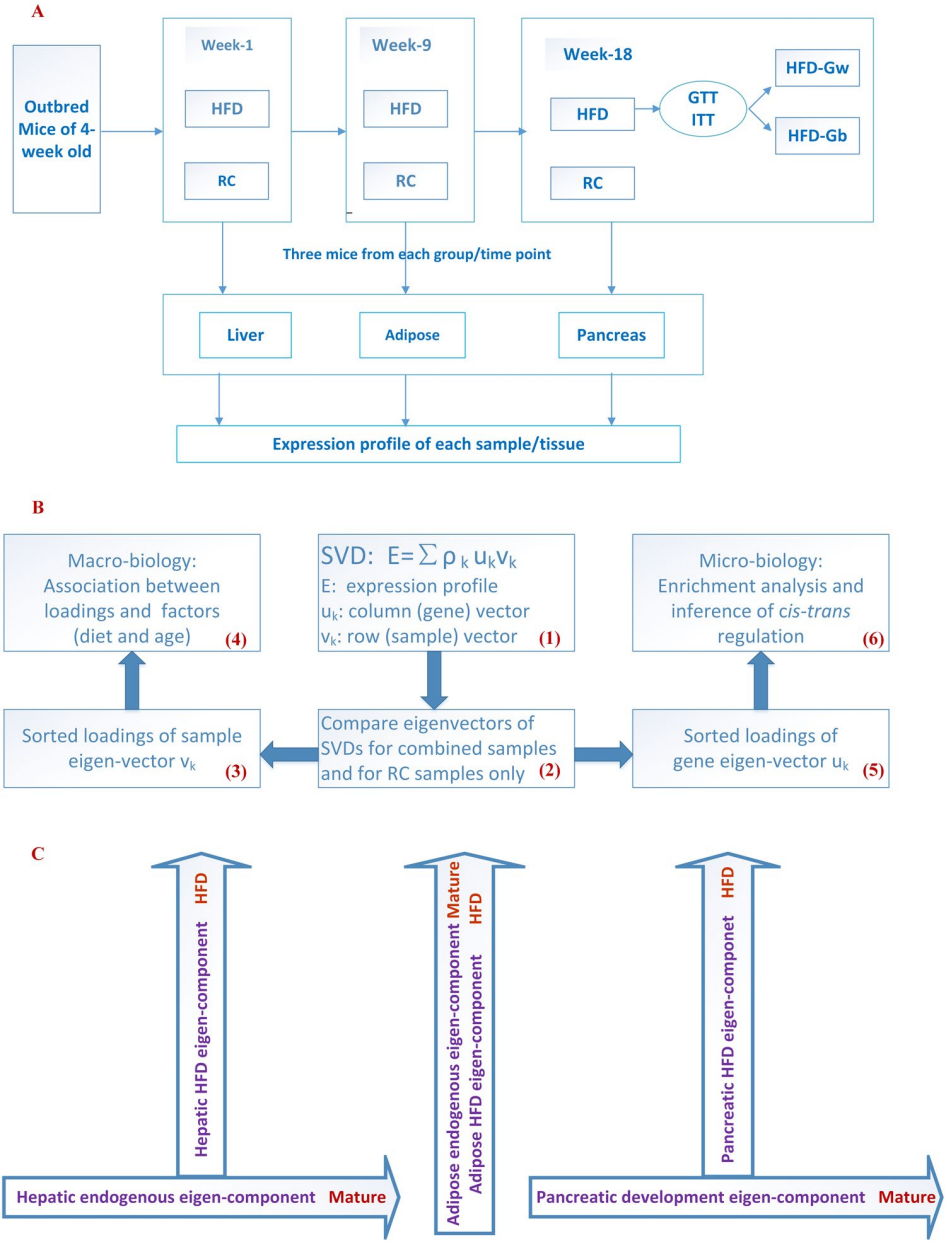
Schemes of the experiment design, dual eigen-analysis and structure of the top eigen-components of the three tissues. (**A**) The outbred mice were fed with RC or HFD. Three mice were randomly selected from each diet group at week 1, 9, and 18. At week 18, the HFD group was further divided into two subgroups, HFD-Gw and HFD-Gb, according to the GTT/ITT results. The expression profiles of their liver, adipose, and pancreas tissues were measured. (**B**) The dual eigen-analysis has several steps. (1) According to the SVD structure, for each tissue, compute the SVD of the expression profile of the HFD and RC samples as well as that for the RC samples only; (2) compare the eigenvectors of the two SVDs and identify the similar and different eigen-components; (3) sort the loadings of each principal sample eigenvector, followed by (4) identifying the associated factor; and (5) sort the loadings of the coupling gene eigenvector, followed by (6) identifying the molecular pathways enriched at its two ends. (**C**) The first and second hepatic eigen-components correspond to the endogenous functional state and the driving impact due to HFD, respectively. Only one meaningful eigen-component was identified for the adipose. The first and second pancreatic eigen-components correspond to the response to the HFD impact and the developmental state respectively.

**Figure 2:**
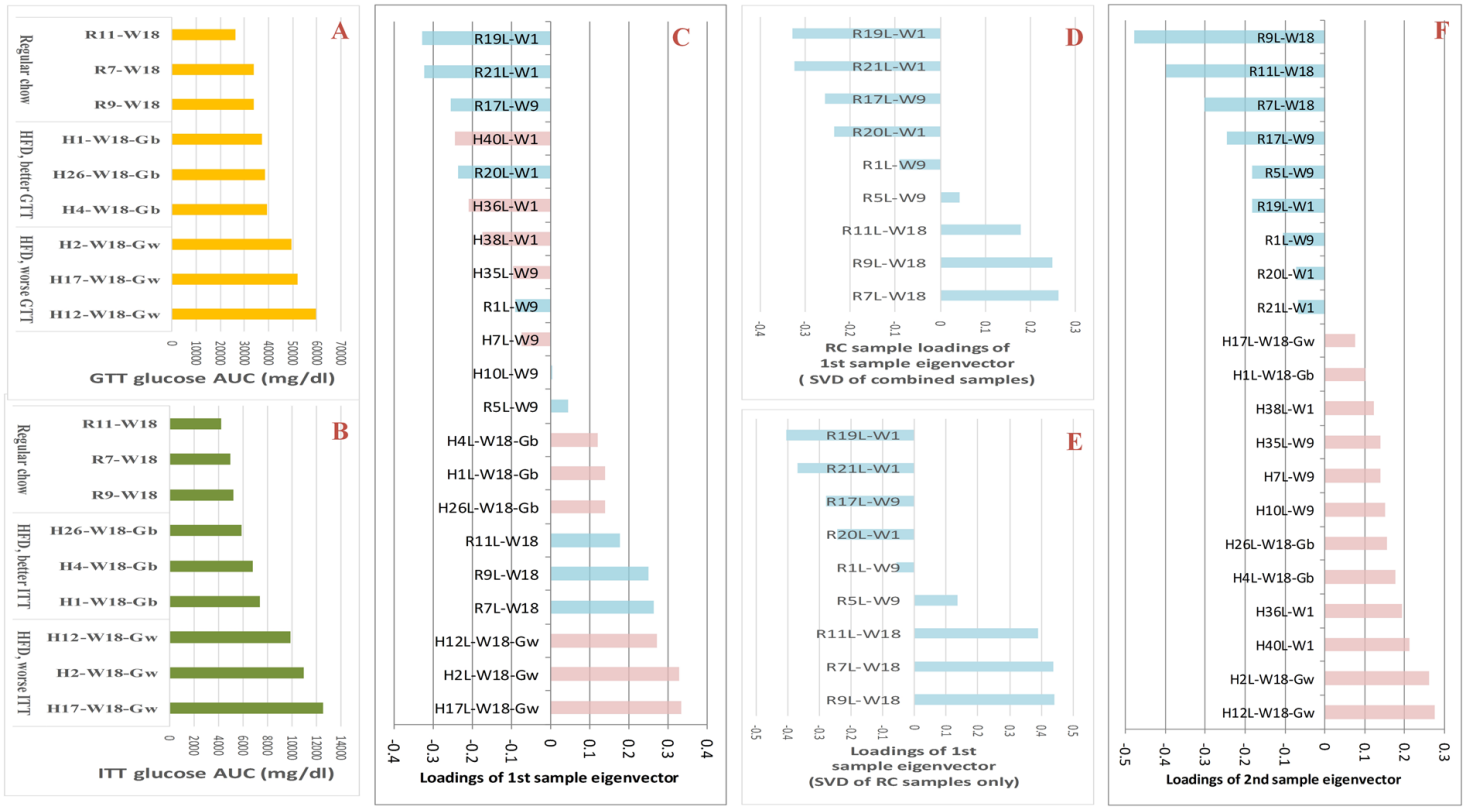
The sorted loadings of the top hepatic sample eigenvectors and their interpretations. (**A**,**B**) The AUC (area under curve) results of GTT and ITT of mouse samples from week 18 were respectively sorted in the ascending order. Shown from top to bottom are the sub-groups of RC, HFD-W18-Gb, and HFD-W18-Gw accordingly. (**C**) The sorted loadings of the hepatic first sample eigenvector from the SVD of the HFD and RC samples. The samples from week 18 were at the bottom. Similar to the ITT (**B**) and GTT (**A**) sorting, the HFD-W18-Gb subgroup located on top of the HFD-W18-Gw subgroup, and even further, on top of the RC samples at week 18. (**D**) Derived from (**C**) by keeping only RC samples. (**E**) When SVD was carried out only for the RC hepatic samples, the sorted loading of its first sample eigenvector displayed a chronological order. The consistency between (**D**) and (**E**) indicates that the first eigen-component reflected an endogenous state of the liver. (**F**) the sorted loadings of the second sample eigenvector from the SVD of the combined samples. The clear-cut separation of the RC and HFD samples indicates that the second hepatic eigen-component reflected the driving impact from HFD.

The sample selection is at the heart of the design of mRNA expression experiments. Three factors: diet, tissue, and time/age, were taken into consideration. The diet factor has two levels: HFD and RC; the tissue factor has three levels: adipose (A), pancreas (P) and liver (L); and the time/age factor has three levels: weeks 1, 9, and 18 from the initiation of the diet treatment. All combinations of these factorial levels lead to 18 cases. The sample size of each group was taken to be three. Figure 1A illustrates the experiment design. Hereafter these groups will be referred to by their factorial level combinations. For example, RC-L-W9 represents the group of regular chow, the liver tissues collected at week 9 when the animals were terminated by sacrifice. Samples are named similarly. For examples, R17P-W9 indicates the pancreas tissue of the RC mouse labeled #17, terminated at week 9; and H4A-W18 indicates the adipose tissue of the HFD mouse labeled #4, terminated at week 18. The sorted glucose tolerance test (GTT) and insulin tolerance test (ITT) results of the samples from week 18 are shown in Fig. 2A-B. Those mice fed with HFD were further divided into two subgroups according to the GTT/ITT results: HFD-W18-Gw (worse GTT/ITT results), and HFD-W18-Gb (better GTT/ITT results). The comparison between these two sub-groups will provide further information regarding the progression of T2D.

The selected samples and their groupings are shown in Table S1. After normalization and summarization of the microarray data, we obtained the expression values of all the mouse genes included in the Affymetrix Mouse430–2.0 Array for each sample. Then we performed typical global analyses on the expression profiles such as hierarchical clustering. The clustering results of the three tissues are quite different (Fig. S1).

### Singular value decompositions (SVD) of expression profiles and dual eigen-analysis

Like clustering, SVD is another approach to summarizing the tissue-specific expression profiles^11^. The top or principal eigenvectors of SVDs are expected to capture the major information in the expression profiles. Table 1 shows the top three singular values of the issue-specific expression profiles and the percentages of their contributions.

**Table 1:** The top three singular values of the three tissue-specific expression profiles and their percentages.

Singular value ranking	Liver		Adipose		Pancreas	
	value	Cumulative percentage	value	Cumulative percentage	value	Cumulative percentage
ρ_1_	1.404	11.46%	2.147	16.06%	1.482	14.77%
ρ_2_	1.092	20.38%	1.165	24.77%	1.244	27.17%
ρ_3_	0.990	28.45%	1.070	32.77%	0.856	35.70%

Each eigen-value/eigen-component is associated with a pair of eigenvectors: one for the samples together with their attributes such as diet and age, and the other for the genes. Each sample eigenvector, denoted by ***ν***, is composed of the weights of all samples, which are referred to as the sample loadings, whereas each gene eigenvector, denoted by ***u***, is composed of the weights of all genes, which are referred to as gene loadings. We proposed a dual eigen-analysis consisting of several parts. First, for a specific tissue, we compute the SVD of the expression profiles of the HFD and RC samples as well as the SVD of the profiles of the RC samples only; second, compare the sample and gene eigenvectors from the two SVDs and identify the similar and different eigen-component; third, sort the loadings of each principal sample eigenvector, and identify the factor of diet or age that is associated with the eigen-component; finally, sort the loadings of the coupling gene eigenvector, and identify the molecular pathways enriched at its two ends. In other words, the sample eigenvectors correspond to the macro-biology information while the gene eigenvectors correspond to the micro-biology information in the dual eigen-analysis. The scheme of the dual eigen-analysis is illustrated in Fig. 1B.

### Sample eigenvectors of tissue-specific expression profiles

**Figure 3:**
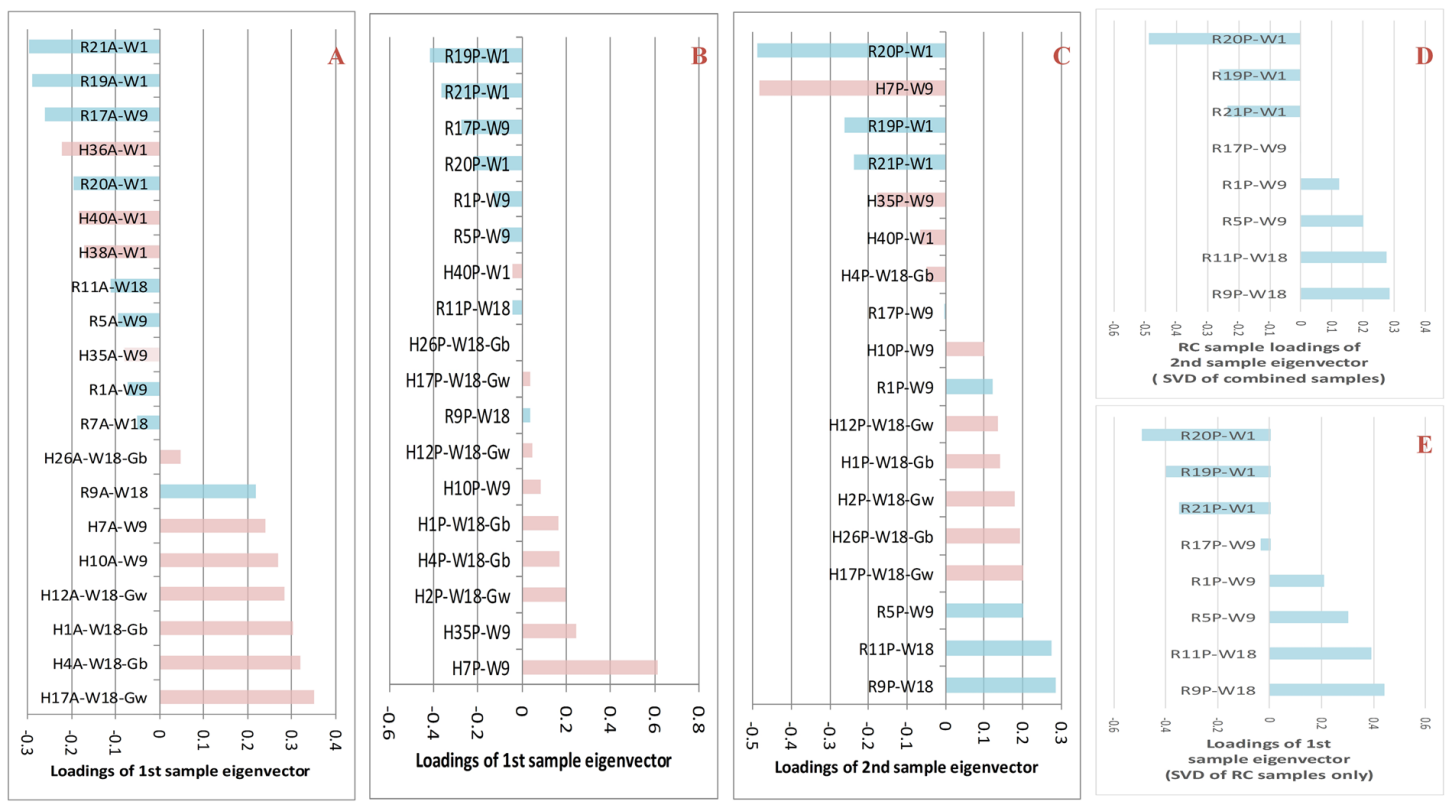
The sorted loadings of the top sample eigenvectors from the adipose and the pancreas and their interpretations. (**A**) The adipose top sample eigenvector ${{\rm{v}}}_{1}$ were highly correlated with the age factor, and after week 9 its correlation with the diet factor becomes more significant. (**B**) The pancreatic first eigenvector ${{\rm{v}}}_{1}$ from the SVD of all RC and HFD samples. Most RC samples were at the top half whereas most HFD samples were at the bottom half. It indicates that the pancreatic first eigen-component reflected predominantly the HFD impact. (**C**) The sorted loading of the pancreatic second eigenvector from the SVD of all RC and HFD samples. (**D**) Derived from (**C**) by keeping only RC samples. (**E**) When SVD was carried out only for the RC pancreatic samples, the sorted loadings of its first sample eigenvector displayed a chronological order. The exact consistency between (**D**) and (**E**) indicates that the pancreatic second eigen-component reflected predominantly the pancreatic development over time.

Following the framework of the dual eigen-analysis, we evaluated statistical associations between the loadings of the principal sample eigenvectors and the factors of diet, age and diabetic phenotypes, see Figs 2–3, and see Supplementary Materials for details. Consequently, we identified the macro-biology meanings of the principal eigen-components of the three tissues, whose structures are illustrated in Fig. 1C.

It is both interesting and intriguing that two significant eigen-components were identified in the expression profiles of the liver and pancreas tissues. In the liver, the first eigen-component represents the hepatic endogenous functional state while the second represents the HFD impact. In the pancreas, the first eigen-component represents the HFD impact while the second represents the stage of the pancreas development. The two orthogonal components were all defined by data themselves. We do need, in this context, a reasonable biological interpretation of orthogonality, which is a general mathematical notion. Under certain technical settings, orthogonality can be interpreted as being uncorrelated. In fact, when expression profiles only from the RC samples were considered, the HFD eigen-component in both liver and pancreas disappeared in the SVD. Hence, the impact imposed by HFD, represented by the HFD eigenvector, can be thought as an exogenous factor that is roughly uncorrelated with the tissue-specific endogenous state represented by their functional or developmental eigen-component respectively in the liver and the pancreas. In short, HFD, as an exogenous factor to the hepatic function or to the pancreatic development, made its impacts on the two tissues indirectly.

In contrast, only one meaningful eigen-component was observed in the adipose expression profiles, of either the pooled samples or the RC samples only. As shown later, down-regulated insulin signaling pathway was significantly indicated in the adipose top gene eigenvector. This suggests that the adipose tissue exhibits, along the age axis, a higher susceptibility to insulin resistance. HFD simply accelerates the progress of this susceptibility.

It is noted that the above results are limited to the mouse samples up to age 23-week-old (5 weeks plus 18-week HFD/RC treatment), and the SVD structure may evolve over a longer time horizon.

### Enrichment analysis of the tissue-specific gene eigenvectors by Wilcoxon scoring

Next we examined the micro-biology embedded in the gene eigenvector ***u*** coupled with each sample eigenvector ***v***. In accordance with a given sample eigenvector, we sorted the loadings of its coupling gene eigenvector and tested what gene subsets were enriched at the two ends. The enrichment analysis was based on the Wilcoxon scoring method that uses the ranks of the gene loadings. It is noted that if a gene subset is up-enriched at one end of an eigenvector, then equivalently it is down-enriched at the other end of the same vector because the ranks of the loadings are just reversed when we change the order of sorting from ascending to descending. Occasionally, we simply use “enriched” instead of “up-enriched” without confusion.

**Figure 4:**
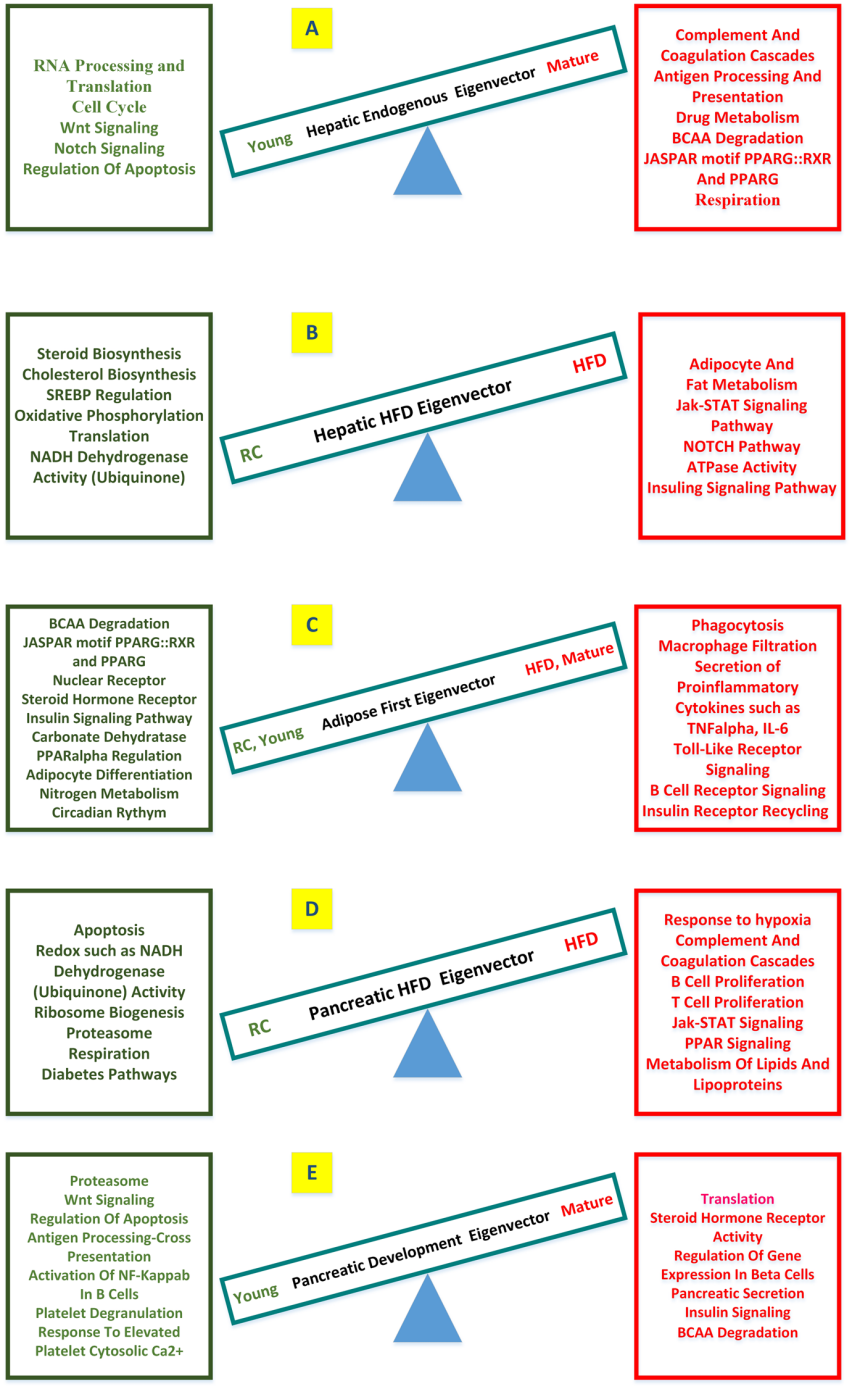
Summarization of the biological processes and molecular functions enriched at the two ends of the top gene eigenvectors. The up-enriched categories are shown on the right in red, whereas the down-enriched ones are shown on the left in green. (**A**) The hepatic first or endogenous gene eigenvector ${{\rm{u}}}_{1}\,$coupled with v_1_ in Fig. 2C. (**B**) The hepatic HFD, or second gene eigenvector ${{\rm{u}}}_{2}\,$coupled with v_2_ in Fig. 2F. (**C**) The adipose first gene eigenvector ${{\rm{u}}}_{1}\,$coupled with v_1_ in Fig. 3A. (**D**) The pancreatic HFD, or first gene eigenvector ${{\rm{u}}}_{1}\,$coupled with v_1_ in Fig. 3B. (**E**) The pancreatic development, or second gene eigenvector ${{\rm{u}}}_{2}\,\,$couplded with v_2_ in Fig. 3C. Notably, the KEGG pathway of valine, leucine and isoleucine degradation were down-enriched at the HFD and mature end of the top adipose eigenvector (**C**) while up-enriched at both the mature end of the hepatic first/endogenous eigenvector (**A**) and the mature end of the pancreatic second/development eigenvector (**E**). The JASPAR motif PPARG::RXR was down-enriched (p-value = 0.0082) at the HFD and mature end of the top adipose eigenvector (**C**) while up-enriched (p-value = 0.013) at the mature end of the hepatic first/endogenous eigenvector (**A**).

We compiled the enrichment analysis in Tables S2–S11 for the hepatic first/endogenous, hepatic second/HFD, the adipose first, and the pancreatic first/HFD as well as its second/development eigenvectors accordingly. We organized the most significant subsets from KEGG^12–14^, Gene Ontology (GO) and REACTOME database into groups. The highlights and details of these enrichment analyses can be found in the section “Details of the enrichment analysis of the tissue-specific gene-eigenvectors by Wilcoxon scoring” of Supplementary Materials. A summary of these enrichment results of the five gene eigenvectors are illustrated in Fig. 4.

### Different patterns of inflammation- and macrophage-related gene expression in adipose and pancreatic tissues

HFD can induce a chronic low-grade inflammatory state associated with obesity^15^. In the process of such inflammation development, macrophage infiltration plays an important role^16^. We observed different patterns of inflammation- and macrophage-related gene expressions in adipose and pancreas in the HFD gene expression eigenvectors of adipose and pancreas, see Supplementary Materials (Table S12–13).

### Down-enriched insulin signaling pathway in the adipose top gene eigenvector

**Figure 5:**
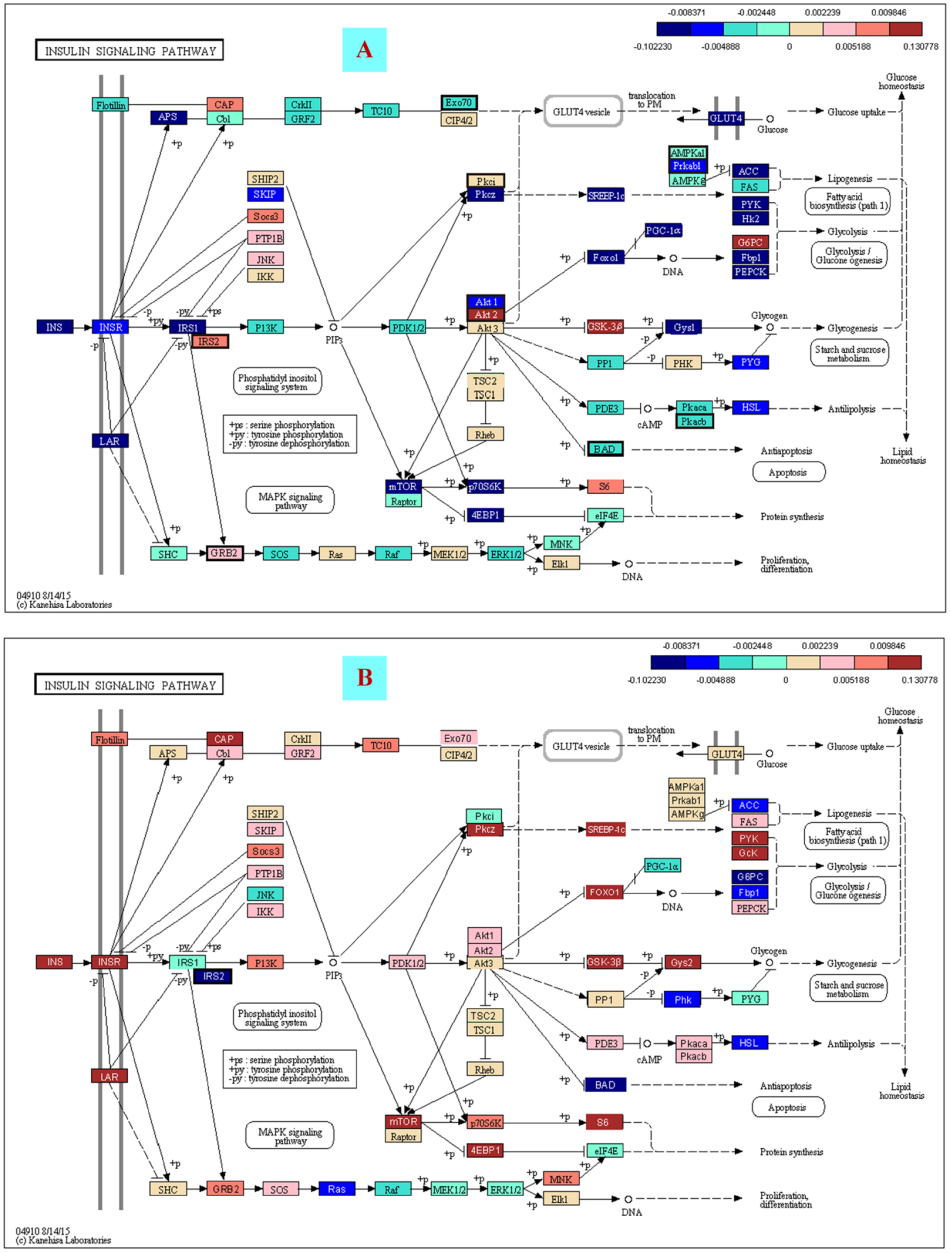
The loadings of the top gene eigenvectors on the KEGG insulin signaling pathway^12^. (**A**) The adipose first gene eigenvector. The down-regulated genes at the HFD and mature end are marked in blue and up-regulated ones are marked in red. The most down-regulated critical genes included *Insr, Irs1, Glut4*, and *PGC-1*. The top gene eigenvector captured the most distinguished pattern of insulin resistance in the adipose induced by HFD. (**B**) The hepatic HFD or second gene eigenvector. In contrast, the hepatic HFD eigenvector did not show an obvious down-regulation pattern.

One of the important pathologic characteristics of T2D is insulin resistance, which is resulted from impairment of insulin signaling pathway. The loadings of the adipose first gene eigenvector were mapped on the KEGG insulin signaling pathway as shown in Fig. 5A, where down-regulation was marked in blue and up-regulation was marked in red. Many critical genes in this pathway were significantly changed. Notably, *Insr* (insulin receptor) and *Irs1* (insulin receptor substrate 1), the two key genes at the top of the cascade were down-regulated. In addition, the expression of three downstream genes: *Hk2* (Hexokinase 2), *Glut4* (Glucose transporter type 4,) and *PGC-1α* (peroxisome proliferative activated receptor gamma, coactivator 1 alpha), which are directly linked to insulin effects, were significantly down-regulated too. The adipose first eigenvector captured the most distinguished pattern of impairment in the insulin signaling pathway at the transcriptional level. In contrast, the hepatic HFD eigenvector did not show much transcriptional down-regulation in the insulin signaling pathway (Fig. 5B and Tables S4–S5).

### Profound down-regulation of cholesterol/steroid biogenesis in the hepatic HFD gene eigenvector

When we checked the hepatic HFD gene eigenvector in details, it turned out that almost all the genes of the cholesterol biogenesis pathway were at the very end of down-regulation. The details of these genes and their *cis-trans* regulation can be found in Supplementary Materials (Table S14 and Figs S4–S5).

### Diverse expression patterns of BCAA degradation pathway and diverse patterns of the PPARγ regulation in the adipose, the liver, and the pancreas

BCAA (leucine, isoleucine and valine) are among nine essential amino acids and have to be supplied from food. The role of BCAA has been implicated in the insulin resistance and insulin secretion. Two definitions of BCAA degradation for comparative analysis were considered in the present study. The Affymetrix microarray includes probe sets corresponding to 39 genes in the valine, leucine and isoleucine degradation pathway provided by KEGG. A core subset of 16 genes comprises the BCAA catabolism pathway of REACTOME.

In the first gene eigenvector of the adipose tissue, the pathway of BCAA degradation was down-enriched at the HFD end. The loadings of adipose first gene eigenvector were mapped on the KEGG BCAA degradation pathway as shown in Fig. S6, where the down-regulated genes were marked in blue, including the important genes Bcat1/2 (branched chain aminotransferase 1 or 2) and Bckdha/b (branched chain ketoacid dehydrogenase E1, α or β polypeptide), and up-regulation was marked in red.

The results were subtler in the liver and pancreas tissue, since we identified two pairs of eigenvectors for each. In the hepatic endogenous eigenvector, the pathway of BCAA degradation was significantly up-enriched at the mature-age end whereas no enrichment pattern was found in the hepatic second/HFD gene eigenvector. In the pancreatic second/development gene eigenvector, the pathway of BCAA degradation was up-enriched at the mature-age end whereas no enrichment pattern was found in the first/HFD gene eigenvector. These results showed different regulation patterns of BCAA degradation in the gene eigenvectors of the three tissues.

The direct comparisons of the HFD and RC groups for different tissues along the time course of the experiments can be found in Table S15. We observed opposite regulations of the BCAA degradation pathway in the adipose and pancreas tissues, namely, down-regulation in the adipose tissue while up-regulation in the pancreas.

Impaired glucose tolerance and insulin resistance as measured by GTT/ITT results were associated with the HFD (Fig. 2A-B), and thus associated with BCAA degradation in the adipose tissue. This association between GTT and down-regulated BCAA degradation pathway in the adipose tissue was further validated by comparing the samples from the HFD-W18-Gw and the HFD-W18-Gb subgroups (Table S15). In comparison, most pairwise comparisons between the two diet groups in the pancreas showed up-regulation at week 18.

The BCAA degradation pathway is associated with PPARγ regulation as it has been reported that the treatment with PPARγ agonist ligand to obese rat modulated BCAA degradation pathway in the adipose tissue. PPARγ, a homolog of PPARα, is a well-known key regulator of adipocyte differentiation both *in vitro* and *in vivo*. According to the transcriptional inference of BASE2.0, see METHODS, the JASPAR motif PPARG composed of a consensus sequence 5′-AGGTCA-3′ followed by its reverse complement as shown in Fig. S7, was enriched (p-value = 0.029) at the down-regulation end of adipose gene eigenvector. The JASPAR motif PPARG::RXRA, which is roughly composed of two consensus sequences 5′-AGGTCA-3′, was enriched (p-value = 0.0082) at the same end. See Fig. S7 for their sequence logos. Its TRANSFAC counterpart PPARDR1_Q2 was enriched as well. It is a binding motif of PPARγ that can form a heterodimer with the retinoid X receptor RXR-alpha.

However, along the hepatic first gene eigenvector, the JASPAR motif PPARG::RXRA was enriched (p-value = 0.013) at the mature-age end. A similar TRANSFAC motif DR1_Q3 was enriched (p-value = 0.0076) in the liver as well.

### A group of genes downstream of HIF-1 signaling pathway up-regulated along the pancreatic first/HFD gene eigenvector in concert with the unordinary expression of adiponectin

With a close look at the sorted loadings of pancreatic HFD gene eigenvector, we found the top gene is *Car3* (carbonic anhydrase 3, see Table S16), which was reported to be a target gene of HIF-1 (hypoxia-inducible factor 1) signaling pathway^17^. Furthermore, a group of genes, which either stimulate or participate in angiogenesis, were identified as downstream of HIF-1 signaling pathway (http://www.kegg.jp/) in the sense that the loadings of either these genes or their homologs have high ranks at the HFD end. Specifically, *Ang* (angiogenin, ribonuclease, RNase A family, 5, rank 19) and *Angptl4* (angiopoietin-like 4, rank 194) correspond to the node ANGPT (angiopoietin) that stimulates angiogenesis. The encoded proteins are involved in mediating reciprocal interactions between the endothelium and surrounding matrix, inhibit endothelial permeability, and are also involved in blood vessel maturation and stability. We also observed *Serpine2* (rank 157), the homolog of *Serpine1* (serine (or cysteine) peptidase inhibitor, clade E, member 1), homologs of *Timp1* (tissue inhibitor of metalloproteinase 1): *Timp4* (rank 25), *Timp2* (rank 130), and *Timp3* (rank 180), which are members of the tissue inhibitor of metalloproteinase family. *Flt1* (VEGFR-2, FMS-like tyrosine kinase 1, rank 946), acts as a cell-surface receptor for VEGFA (vascular endothelial growth factor A) and plays an essential role in the regulation of angiogenesis. *Egr1* (early growth response 1, rank 37) is a zinc finger transcription factor involved in a number of early responses to stimuli including hypoxia and epidermal growth factor (EGF), which plays a role of angiogenesis stimulation in the KEGG HIF-1 signaling pathway. Besides, *Efemp1* (epidermal growth factor-containing fibulin-like extracellular matrix protein 1) ranked 49 and *Eps15* (epidermal growth factor receptor pathway substrate 15) ranked 439. As a matter of fact, the GO biological processes – Positive Regulation of Angiogenesis and Vasculogenesis – were significantly up-enriched at the HFD end even after Bonferroni correction, See Table S8. Moreover, *Cav1* (caveolae protein 1, rank 62) is the primary negative regulatory protein for endothelial nitric oxide synthases (eNOS), and is directly activated by HIF-1^18, 19^. Another gene downstream of the KEGG HIF-1 signaling pathway, *Trf* (transferrin, rank 10) is an iron binding transport protein that is responsible for the transport of iron from sites of absorption and heme degradation, to those of storage and utilization. Related to iron metabolism, *Hp* (haploglobin) ranked 15, and hemoglobin adult chains *Hba-a1* (hemoglobin alpha, adult chain) and *Hba-b1* (hemoglobin, beta adult chain) ranked 48 and 109 respectively. Other high-ranking genes stimulating angiogenesis include *Fgf1* (fibroblast growth factor 1) and its receptor *Fgfrl1*, transforming growth factor *Tgfbi* (transforming growth factor, beta induced) and its receptors *Tgfbr2, Tgfbr3*. All these genes relating to HIF-1 signaling pathway and other genes relating hypoxia according to GO annotation, which are within top 1000 loadings, are listed in Table S16.

**Figure 6:**
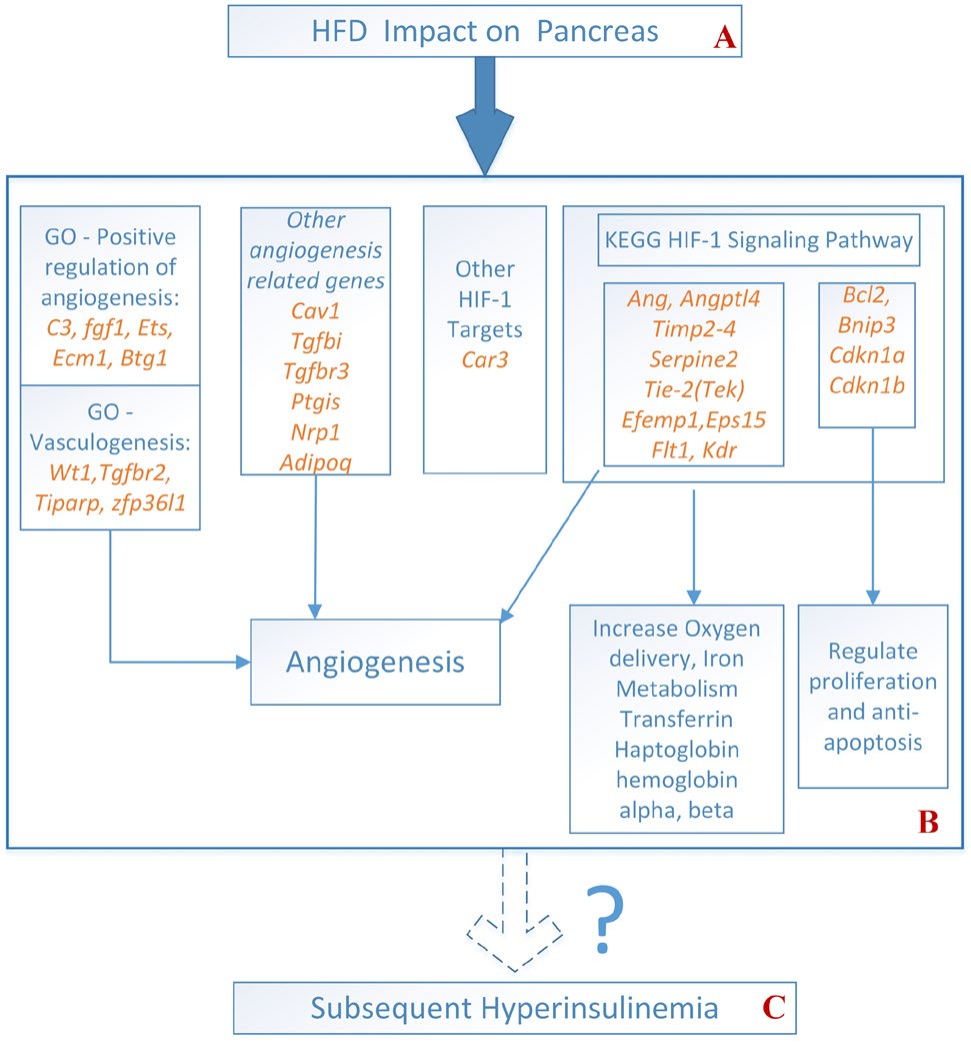
A number of genes including those downstream of HIF-1 signaling pathway were up-regulated in the pancreas in response to HFD. The sorted loadings of pancreatic HFD gene eigenvector demonstrated that the exposure to HFD (**A**) upregulated the genes downstream of the KEGG HIF-1 signaling pathway (**B**) Most of these genes are related to increasing oxygen delivery including angiogenesis whereas some others are related to regulation of proliferation and anti-apoptosis. Genes related to angiogenesis other than those in the KEGG HIF-1 signaling pathway such as *Ang* were also examined, including *Adipoq*, *Cav1* and *Ptgis*, and others from the Biological Processes of Gene Ontology (GO): Positive Regulation of Angiogenesis and Vasculogenesis. The top ranked gene *Car3* in the HFD gene eigenvector was reported to be a target of HIF-1signaling pathway^17^. (**C**) Whether this kind of up-regulation attributed, at least in part, to the subsequent over-secretion of insulin from the pancreas that results in hyperinsulinemia is worth of future investigations.

Notably, the loading of adiponectin ranked 3 in the pancreatic HFD gene eigenvector. Our previous report confirmed that almost no expression of adiponectin was detectable in the pancreas of control mice fed with RC, but an aberrant expression of adiponectin was found from the pancreatic vascular endothelial cells (VECs) of mice fed with HFD^6^. Here the timing indicated from the loadings of the pancreatic first samples eigenvector, in which the HFD samples from week 9 stood at the bottom and the HFD samples from week 18 stood in the middle (Fig. 3B), was highly consistent with the previously reported timing of the unordinary adiponectin expression in pancreatic VECs of HFD-fed mice, namely, detectable as early as week 1 after starting the HFD, reached its highest level at week 9 and then declined at week 18. In the previous report, hyperinsulinemia was observed in HFD fed-mic at week 18. In this study with limited samples at week 18, a trend of hyperinsulinemia in HFD fed-mice existed (Fig. S8). The timing suggested that hyperinsulinemia is preceded by the regulation of HIF-1 signaling pathway and particularly its downstream angiogenic functions. All these observations are summarized in Fig. 6.

## Discussion

The primary analysis of this study is based on the strata resulted from the SVD structure of the expression profiles, even though some direct comparative results of samples stratified by diet or age levels were reported as well. The SVD analysis fits the outbred model, in which samples’ genetic backgrounds were not homogeneous and the sample size of each diet group at each time point is at most three. In the SVD, each sample eigenvector pairs up with a gene eigenvector. The macro-biological meaning of a component was identified by the association between the sample loadings and their attributes such as diet and age. The coupled micro-biology, namely, the molecular mechanisms, was identified by inferences such as enrichment analysis based on the gene loadings.

In this report, making use of the dual eigen-analysis, we identified diverse tissue specific characteristics of the expression profiles. BCAA degradation is such an example. It was reported that valine, leucine and isoleucine degradation pathway was up-regulated in the adipose tissue of Zucker Rats when treated with the PPARγ ligands^20^, which also improved insulin sensitivity. For human samples, Shah and colleagues^21^ used the mass spectrometry technique to profile 60 metabolites and found that a cluster of metabolites including BCAA can predict improvement of insulin resistance independent of weight loss. The connection between insulin resistance and BCAA metabolism has attracted much attention recently^22–24^. Using the dual eigen-analysis, we discovered different regulation patterns of BCAA degradation in the three tissues. On one hand, consistent with the earlier report^20^, this pathway was down-regulated at the HFD and mature-age end in the adipose tissue, implying less BCAAs were catabolized under HFD. On the other hand, in the liver and the pancreas, this pathway was up-regulated at the mature-age end along the endogenous gene eigenvector, implying more BCAAs were catabolized. To our knowledge, this is for the first time to report the diverse regulation patterns of BCAA degradation among these tissues. Its implication in the development of T2D needs further investigation.

Related to BCAA metabolism, insulin sensitivity has been reported to be associated with the regulatory role of PPARγ^20^. Thiazolidinediones (TZDs) are PPARγ agonists, which are used to treat T2D by improving insulin sensitivity. Interestingly, similar to the case of BCAA metabolism, in this study we found diverse patterns of PPARγ regulation in the adipose and liver tissues, in the context of HFD. The regulation of PPARγ appeared reduced in the adipose, yet increased in the liver. Such diversity was unveiled for the first time, and it depicts a more comprehensive picture for better evaluation of the effects and side-effects of therapeutic means including current as well as future ones. The side effects of TZD, such as the known hepatic toxicity^25^, are a complicated topic, and are not completely clear. The diversity of tissue-specific PPARγ regulation provides a new angle to evaluate TZD treatment of T2D, and it is worthwhile conducting further investigation in the future.

Increasing evidences reveal that a chronic low-grade inflammatory state plays an important role in the onset and development of T2D. The results from our dual eigen-analysis were consistent with this model. Moreover, from the results of significant up-enriched pathways relating to immune systems and inflammation, we found that following HFD treatment, inflammation occurred in the pancreas as well. Although the gene expression profiles from the adipose and the pancreas demonstrated that both had remarkable inflammatory responses, their mechanisms in the two tissues were somehow different as suggested by this study. In particular, the loading of *Mmd* ranked 28 in the pancreatic HFD gene eigenvector, whereas it was not significant in the adipose eigenvector. *Mmd* has been reported^26^ to be highly expressed in mature macrophages but absent in monocytes. It was also reported that overexpression of *Mmd* in macrophages will increase the production of TNF-α and NO upon lipopolysaccharide (LPS) stimulation^27^. Accumulating evidence has been in support of the concept of local pancreatic islet inflammation as a mechanism of β cell failure in T2D^28^. In this study we contributed one more support via the dual eigen-analysis of the pancreas and adipose expression profiles, even though our outbred mouse model of T2D appeared to still be in the β cell compensation state.

An interesting discovery of this study is that a group of angiogenic genes as downstream of HIF-1 signaling pathway were up-regulated along the pancreatic HFD gene eigenvector, and they were in concert with the unordinary expression of adiponectin. According to the sorted loadings of the gene eigenvector, a large portion of the top genes, which are downstream of HIF-1 signaling pathway, can serve as the readouts of the HIF-1 activity, and the majority of them are related to angiogenesis. A good number of the top loading genes are hypoxia-related as shown by the annotations of GO -biological process, even though it is unclear whether hypoxia really occurred or not. Gunton’s group^29^ has reported that HIF-1α is required for normal pancreatic β cell function, and its decreased level could contribute to β cell dysfunction of T2D. This led us to postulate that along the time course of HFD in this study, a compensatory mechanism may occur in the pancreas via the upregulated HIF-1 signaling pathway, mainly mediated by its downstream angiogenic genes, or in other words, it is very possible that the angiogenic consequences from this group of genes may play a mediator role between the effect of HIF-1 signaling pathway and insulinogenesis. This was not addressed in the report of Gunton’s group^29^. According to the sorted loadings of the sample eigenvector, the compensation was most prominent in samples from week 9, and the strength diminished in samples from week 18. This timing is highly consistent with our earlier report^6^ on the expression of adiponectin in the pancreatic VECs of HFD-fed mice, using quantitative real-time polymerase chain reaction (qPCR) and Western-Blot analyses. These results provided critical validation of the current results based on microarrays. In this study, the whole-genome expression profiles unveiled a more comprehensive picture of the compensatory mechanism.

It is noted that the models used by Gunton’s group and by us have some differences. First, in that study, they specifically deleted HIF-1α in β cells of C57BL/6 mice, and enhanced its regulation through iron chelation with deferoxamine or deferasirox. In contrast, in this study the regulation of HIF-1 activity was read out by its downstream gene expressions in the context of the whole pancreas tissue. Second, the Balb/c and C57BL/6 mice used by Gunton’s group were inbred strains while our CD1 mice were outbred strain which more closely mimicked the polygenic factors for T2D onset in human being. Third, our outbred mice were exposed to HFD for up to 18 weeks, and thereby progressive effects of HFD can be observed. The C57BL/6 mice with β cell–specific HIF-1α disruption exhibited glucose intolerance which developed more severe on a HFD. In our model, the glucose intolerance of HFD-treated mice as measured by GTT was more serious in week 18 than in week 9^6^. Meanwhile, the compensation from the HIF-1signaling pathway is more prominent in week 9 than that in week 18, as shown in this report. Gunton’s group reported that decreased HIF-1α levels in pancreatic islets of T2D could contribute to insufficient insulin secretion of β cells. In our previous and present studies, we observed hyperinsulinemia or a trend of hyperinsulinemia in the outbred mice fed with HFD for 18 weeks. Whether this is a result of the pancreas to counteract or compensate the impairing effects of the HFD at relatively early stage of T2D development, and the connection between this over-secretion of insulin and the upregulated HIF-1 signaling pathway particularly its downstream angiogenic functions, await further investigations.

Another discovery is the profound down-regulation of cholesterol/steroid biogenesis in the hepatic HFD gene eigenvector. This echoed the report that the KEGG pathway of steroid hormone biosynthesis was significantly dysregulated in the liver and adipose in the Goto-Kakizaki rat^5^. In fact, steroid hormones are derived from cholesterol. However, we did not further elaborated on the biological implications of this issue and other results obtained from the dual eigen-analysis. Rather, we provide these interesting computational insights from the dual eigen-analysis as clues for further investigations of T2D.

## Methods

### Experimental animals

Mouse procedures were the same as previously reported^6^. All experimental procedures were approved by the Animal Ethics Committee of Peking University Health Science Center, and all experiments were performed in accordance with the institutional guidelines and regulations. Male outbred CD-1 mice at 3 weeks of age were purchased from Charles River Laboratories via Vital River Laboratories (VRL, Beijing, China) and kept in a temperature-controlled environment. Food and water were available ad libitum unless otherwise indicated. They were used in this study at 4 weeks of age (weighing 15.3 ± 0.6 g) and this time was set as week 0 of the experiment that was then carried out for 18 weeks in total. All mice were randomly assigned to a control group fed on RC (approximately 17% fat, 19% protein, and 64% carbohydrate) and an HFD group fed on the HFD (approximately 42% fat, 19% protein, and 38% carbohydrate). Weeks 1, 9, and 18 were set as time points for performing GTT and ITT. D-gucolse (2 g/kg) for GTT and porcine insulin (1 unit/kg) for ITT were intraperitoneally injected, respectively.

### Sample collection and microarray experiment

Several mice from the RC and HFD groups at the three time points were randomly selected (Table S1) and sacrificed after saline perfusion. The epididymal fat pads, the liver, and the pancreas were collected immediately, and picked up by Capital Corporation http://www.capitalbio.com/ for mRNA extraction and microarray experiments following the standard protocol given by Affymetrix. The raw microarray data were deposited at http://www.ncbi.nlm.nih.gov/geo/ (accession number: GSE77943).

### Preprocessing of microarray data

Each probe set of the Affymetrix mouse430 microarray contains 11 probes, and thus we need to summarize their information into one value for each sample. From the microarray data we seek for differentially expressed genes among the samples. However, the information of gene expression differences is confounded with variations due to uncontrollable experiment factors. To reduce the unwanted variations, we need to normalize the data in one way or another. The microarray data in this study were preprocessed by several methods such as RMA^30^ and MAS 5.0 (http://www.affymetrix.com). It was shown that spatial variations of microarrays due to factors such as uneven hybridization and washing accounted for a large portion of unwanted variations. Thus we applied a more sophisticated preprocessing, namely, the sub-sub normalization^7^ followed by the probe-treatment-reference summarization^8^, to the microarray data of each tissue. Specifically, this procedure normalized each target microarray against a reference as follows. Each Mouse430–2.0 chip, consisting of 1002 by 1002 probes, was decomposed into subarrays of 50 by 50 probes. For each corresponding pair of subarrays on the reference and target chip, a piecewise (with one knot) linear relationship was estimated using least trimmed squares, and was used to adjust the target sub-array. We allowed adjacent subarrays to overlap by 25 probes both horizontally and vertically. Therefore, a probe belonging to multiple subarrays got multiple adjusted values, and we took their average. For each tissue, we selected several raw microarrays as references, and used the probe-treatment-reference model, to summarize the normalized microarrays.

### SVD of gene expression profiles

The log-scale gene expression profile from one tissue was denoted by a matrix $E=[{E}_{ij}]$, where $i=1,\cdots \,g$, and $j=1,\cdots ,s$, *g* is the total number of genes or probe sets, and *s* is the number of samples. The singular value decomposition has the following representation:

$$E=\sum _{{\rm{k}}=1}^{{\rm{s}}}{{\rm{\rho }}}_{{\rm{k}}}{{\rm{u}}}_{{\rm{k}}}{{\rm{v}}}_{{\rm{k}}}^{{\rm{T}}},$$

where ${{\rm{\rho }}}_{{\rm{k}}}$ are nonnegative, decreasing singular values, and their corresponding gene eigenvector ${{\rm{u}}}_{{\rm{k}}}$ and sample eigenvector ${{\rm{v}}}_{{\rm{k}}}$ are respectively of size *g* and *s*, ${{\rm{u}}}_{{\rm{k}}}$ are mutually orthogonal and so are ${{\rm{v}}}_{{\rm{k}}}$. We note the SVD is not unique in the sense that we can change the signs of the components of both ${{\rm{u}}}_{{\rm{k}}}$ and ${{\rm{v}}}_{{\rm{k}}}$ simultaneously without changing the product ${{\rm{u}}}_{{\rm{k}}}{{\rm{v}}}_{{\rm{k}}}^{{\rm{T}}}$. In our analysis, once we set the sign of the sample eigenvector ${{\rm{v}}}_{{\rm{k}}}$ by a macro-biology interpretation, the sign of the gene eigenvector ${{\rm{u}}}_{{\rm{k}}}\,$is determined accordingly.

### Inference of *cis-* and *trans-*regulation by BASE 2.0

The binding *of trans-*acting factors to their *cis-*acting DNA elements near the target genes controls the transcription from DNA to messenger RNA. In functional genomics, it is of great importance to identify the *cis-*elements and *trans-*factors that are involved in the transcriptional program of a given expression profile. In the current study we adopted the method BASE 2.0^31^ to infer the *cis-* and *trans-*regulation. The method searches the maximum association between a bind affinity profile of a certain *cis-*element and the expression difference profile along the direction of sorted expression differences. The bind affinity profile of a *cis-*element was determined computationally. That is, we considered those *cis-*elements whose position weight matrices were provided by the databases TRANSFAC and JASPAR. Then the occurrences of a *cis-*element were searched in the upstream region of each gene according to its position weight matrix using the software MAST and a transformed version of the significance was taken to be the binding strength. Details of the current version BASE 2.0 can be found in the earlier report^31^.

### Data Availability

The raw microarray data were deposited at http://www.ncbi.nlm.nih.gov/geo/ (accession number: GSE77943). A reviewer link has been generated as follows:

https://www.ncbi.nlm.nih.gov/geo/query/acc.cgi?token=afwrmwuixtktbib&acc=GSE77943

## Electronic supplementary material

Supplementary Materials
