# Supplementary material for: A Novel Dual Eigen-Analysis of Mouse Multi-Tissues’ Expression Profiles Unveils New Perspectives into Type 2 Diabetes
Source: Sci Rep. 2017 Jul 11;7:5044. doi: 10.1038/s41598-017-05405-x (PMC5506042; doi:10.1038/s41598-017-05405-x)
Supplement: Supplementary file 1 — Supplementary Materials [file 41598_2017_5405_MOESM1_ESM.pdf]

Supplementary Materials

A Novel Dual Eigen-Analysis of Mouse Multi-Tissues' Expression Profiles Unveils New Perspectives into Type 2 Diabetes

Authors: Lei M Li, Xiuxiu Liu, Lin Wang, Yong Wang, Xiuqin Liu, Xue Tian, Fuzhou Gong, Li Shen, Xiao-ding Peng

Sample Selection

The selected samples are shown in Table S1. The sample H36P did not pass the quality check for the microarray experiments and was dropped. After the microarray experiments were finished, it turned out that the quality of H38P, R7P and H2A are not as good as others, and thus were excluded in the report.

Table S1: Mouse samples selected to the microarray experiments.

	Week 1	Week 9	Week 18	
RC	R19, R20, R21	R1, R5, R17	R7, R9, R11	
HFD	H36, H38, H40	H7, H10, H35	H26, H1, H4 (better GTT results)	HFD-W18-Gb group
			H17, H12, H2 (worse GTT results)	HFD-W18-Gw group

Hierarchical clustering analysis of gene expression data

One routine analysis of microarray data is clustering. Since the expression profiles were substantially different across the three tissues, we carried out clustering for samples of each tissue as well as for all samples across tissues. Specifically, the median absolute deviation (MAD) of each gene was calculated and the genes with the largest 10000 MADs were chosen as the top 10000 most informative genes. Next we used TreeView and Cluster [1,2] to implement the bi-directional hierarchical clustering based on average linkage.

We clustered the expression profiles of each tissue separately and the tissue-specific clustering results of the samples are shown in Fig S1 A-C. First, in the liver tissue clustering, samples from week 1, week 9 and week 18 are roughly and respectively clustered together and then within each week the samples are clustered by the diet.

The sample H26L-W18, though from week 18, showed good GTT and ITT results, and were indeed clustered next to mice H35L-W9, H7L-W9 and H10L-W9, which are from week 9. Second, in the adipose tissue clustering, the samples of adipose tissue from week 1 are next to one another, but the HFD and RC group are not separated. After week 9, samples were clustered primarily by diet. Third, in the pancreas tissue clustering, R17P-W9 and R1P-W9, two RC samples from week 9 are next to the samples from week 1. Other samples after week 9 forms three clusters, one consisting of H4P-W18, H2P-W18, H1P-W18, H7P-W9, H35P-W9, one consisting of H26P-W18, H17P-W18, H10P-W9, H12P-W18 and one including R5P-W9, R11P-W18, R9P-W18. In summary, HFD makes different kinds of influences on the expression profiles of the three tissues.

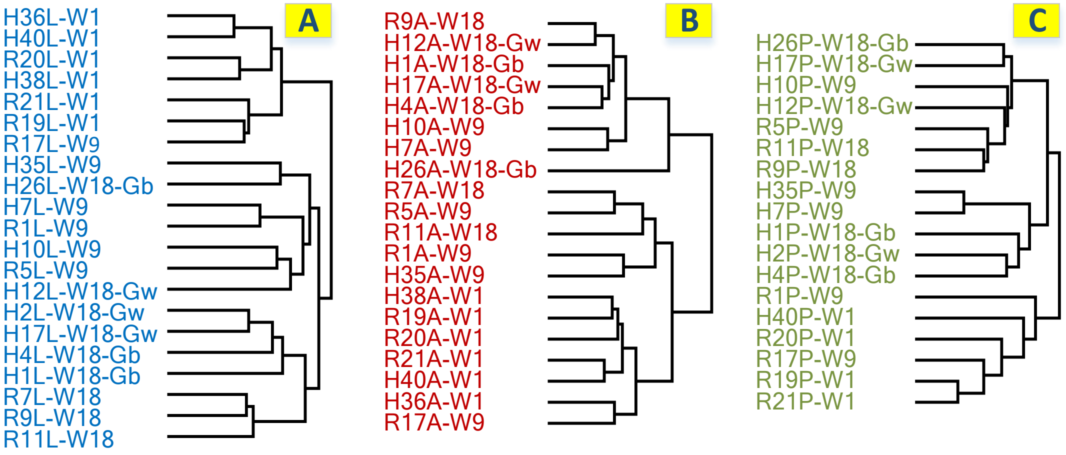

Fig. S1: Hierarchical clustering of gene expression profiles of each tissue. Only the sample clustering results are shown**.** (A) The clustering of the liver samples; (B) The clustering of the adipose samples; (C) The clustering of the pancreas samples. The expression data were centerized by removing the median of each gene across all samples.

SVD of expression profiles.

SVD is a powerful dimension-reduction method that enables us to extract useful information from the fairly large matrices of expression values. The top eigenvalue and eigenvectors of each tissue-specific SVD are essentially the adjusted averages, and these baselines provide no interesting biological explanation to the expression variations. Hence we exclude this baseline component when we refer to the first and second eigenvalues or eigenvectors.

Sample eigenvectors of tissue-specific expression profiles.

Liver: The sorted loadings of the hepatic first sample eigenvector are shown in Fig. 2C. Age is the primary factor that is consistent with the sample sorting. Specifically, all samples from week 18 were at the bottom, while samples from week 1 and week 9 were mixed from top to middle. A close look at the samples from week 18 showed an interesting pattern: similar to the ITT (Fig. 2B) and the GTT (Fig. 2A) sorting, the HFD-W18-Gb subgroup located on top of the HFD-W18-Gw subgroup, and even further, on top of the RC-W18 group (Fig. 2C). Most samples at week 9 were in the middle except R17L-W9. The clustering of the loadings unraveled deeper and cleaner meanings than the hierarchical clustering pattern shown in Fig. S1 did.

The hepatic second sample eigenvector, which is mathematically orthogonal to the first one, made a perfect cut between the HFD and the RC group, regardless the age and GTT factors (Fig. 2F). That is, in its sorted loadings, all negative values corresponded to the RC group, and all positive values corresponded to the HFD group. Such a perfect separation of the HFD and RC mice is almost impossible an event of chance. If we represent an RC sample by a white ball and a HFD sample by a black ball, then the above event is equivalent to drawing 9 balls from a box consisting of 9 white ones and 12 black ones. Under the random setting, the chance of the event that all selected balls are white is only 3.4E-06.

The sorted loadings of the hepatic first and second sample eigenvectors led us to postulate that the first principal component represents, by and large, the endogenous metabolic and functional state of the liver tissue whereas the second component represents, by and large, the driving force from HFD. To test this hypothesis, we carried out SVD on the expression profiles consisting of the RC samples only. The sorted sample loadings of its first sample eigenvector are shown in Fig. 2E, which displayed a chronological order. Except for the swap of R7L-W18 and R9L-W18, the sorting was almost identical to that of the RC sample loadings of the first sample eigenvector of combined samples shown in Fig. 2D, which is derived from Fig. 2C. In fact, the loadings of R7L-W18 and R9L-W18 almost tied in both cases. Next we considered their corresponding gene eigenvectors. The scatter plots of the loadings of the first gene eigenvectors from the RC group only versus those from the combined group is shown in Fig. S2. Their Pearson correlation is 0.86. Put together, these results do support our hypothesis regarding the interpretation of the top two hepatic principal eigen-components. Hereafter the first eigen-component will be referred to as the hepatic endogenous eigen-component while the second will be referred to as the hepatic HFD eigen-component.

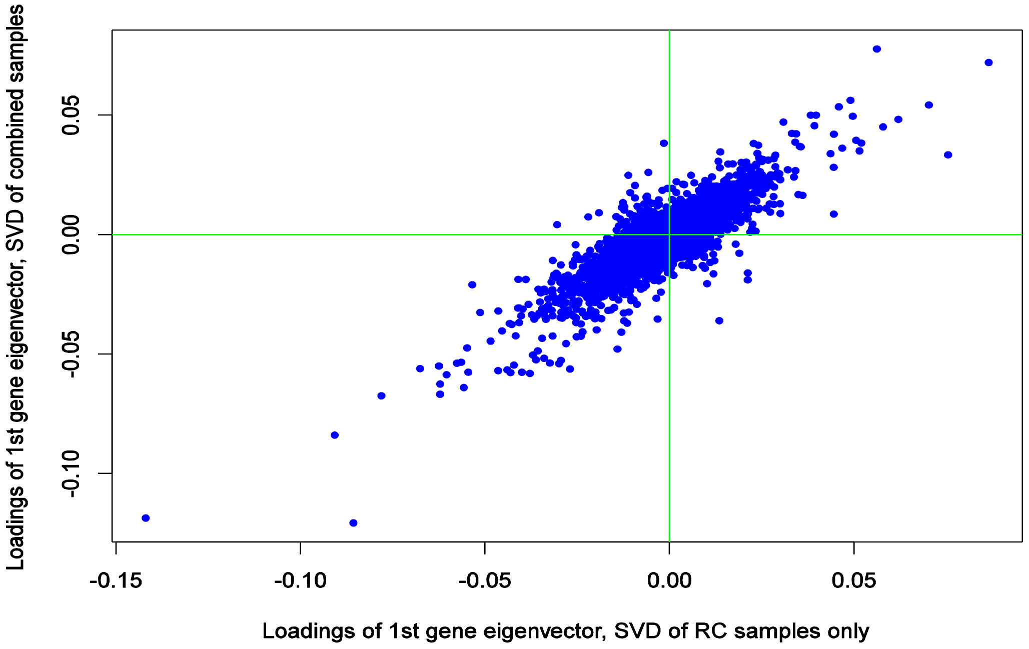

Fig. S2: The scatter plot of the loadings of the first gene eigenvector from the SVD of the RC samples only versus the loadings of the first gene eigenvectors from the SVD of the combined samples (Liver tissue). The Pearson correlation between them is 0.86.

Adipose: The adipose tissue expression profile, unlike the hepatic profile, was well approximated by its first eigen-component, whose singular value was 2.15 compared with the second value 1.17, which was close to the third one 1.07. The sorted loadings of the first sample eigenvector are shown in Fig. 3A. They were highly correlated with the age factor, and after week 9 the correlation with the diet factor becomes more noticeable. Nevertheless, the sorting pattern was somewhat different from that of the first hepatic sample eigenvector. In particular, an RC sample R9A-W18 from week 18 located on top of an HFD sample H35A-W9 from week 9. Moreover, the two HFD subgroups from week 18, respectively with worse and better GTT results, could not be separated by the adipose first sample eigenvector as by the first hepatic eigenvector.

Pancreas: The pancreas, whose islet cells produce and secret insulin, is the tissue critical for the understanding of T2D development. Like the liver tissue, no dominating singular value stood out. In fact, the top two values 1.48 and 1.24 contributed to 27.2% of the total singular values, compared to 20.4% in the liver tissue. The sorted loadings of its first sample eigenvector v_1 are shown in Fig. 3B. Most RC samples were at the top half whereas most HFD samples were at the bottom

half. When we excluded the HFD samples, the RC samples were well ordered by their ages except the swap of R20P-W1 and R17P-W9. However, the rankings of the HFD samples were subtler. The two samples standing at the very bottom were H7P-W9 and H35P-W9, which were from week 9. Meanwhile, the samples H17P-W18-Gw and H12P-W18-Gw from the week 18 located in the middle. Most other HFD samples were between them. This patter was not similar to what have been observed in the liver and adipose tissue. One explanation to this phenomenon is that a compensatory mechanism initiated in the pancreas around week 9 when the stress of HFD reached to a certain point, and the compensation diminished over time.

While the first component primarily corresponded to the stress of HFD on pancreas, the RC sample standings spread out in the sorted loadings of the second sample eigenvector v_2 as shown in Fig. 3C. Fig. 3D was derived from Fig. 3C by keeping only RC samples, which displayed a perfect chronological order. Similar to the analysis of the liver tissue, we carried SVD on the pancreatic expression profiles consisting of the RC group only. The sorted loading of its first sample eigenvector are shown in Fig. 3E. The exact consistency between Fig. 3D and Fig. 3E indicates that the pancreatic second eigen-component reflected predominantly the pancreatic development over time. Next we switched to their coupled gene eigenvectors. The scatter plot of the loadings of the first gene eigenvector from the RC group versus those of the second gene eigenvector from the combined group is shown in Fig. S3. The Pearson correlation is 0.80, and the results provide furtherl support to the hypothesis that the second principal component represents, by and large, the development status of the pancreas tissue whereas the first represents the HFD impact.

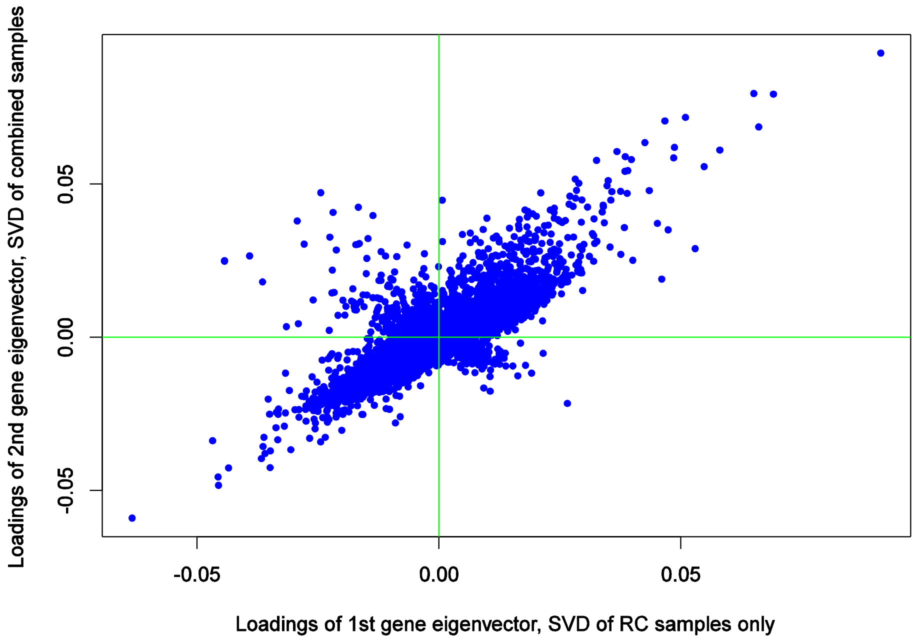

Fig. S3: The scatter plot of the loadings of the first gene eigenvector from the SVD of the RC samples only versus the loadings of the second gene eigenvector from the SVD of the combined samples, the RC and HFD group (pancreas tissue). The Pearson correlation between them is 0.80.

Wilcoxon rank sum scoring of the gene subsets defined by KEGG pathways, GO, and Reactome.

One of our goals was to deduce the cellular activity changes in the mice fed with HFD from the expression profiles. After data preprocessing, we could directly calculate the vector of all gene expression differences between a specific pairs of samples to be compared, or could obtain the gene loadings of a principal eigenvector from the SVD of a tissue-specific expression profile. Sorting any one of these kinds of gene vectors in the ascending or descending order resulted in a gene spectrum. In this report a common theme of analyzing these spectra is the enrichment analysis based on gene subsets defined by bioinformatic database. In this study, we considered the following gene subsets: KEGG pathways (Release 58.0), GO annotation gene sets, classified according to biological processes, molecular functions, and cellular components, and the Reactome database (Version 40). Then we carried out the enrichment analysis using the Wilcoxon scoring method proposed earlier [3].

Enrichment analysis of expressions is a typical computational method to interpret the data by functional gene subsets or annotations. In this report enrichment analysis of each gene eigenvector was carried out based on the gene loadings, but not directly on

the gene expression values. In consideration of robustness with respect to the scale issue, we took the rank-based Wilcoxon scoring method as the tool for enrichment analysis. The method was proposed in [3] and its applications to the yeast aging project have led to new biological discoveries [4,5]. A recent application of Wilcoxon scoring method has also been reported [6]. The functional modifications of the molecular landscape imposed by HFD are summarized in Fig. 4.

We compiled the enrichment analysis in Tables S2-S11 for the hepatic first/endogenous and its second /HFD, the adipose first, and the pancreatic first /HFD as well as its second /development eigenvectors accordingly. Both p-values and Bonferroni corrections for multiple testing were evaluated (see the last two columns of each table). We organized the most significant subsets from KEGG, Gene Ontology (GO) and REACTOME database into groups.

Details of the enrichment analysis of the tissue-specific gene-eigenvectors by Wilcoxon scoring.

Hepatic first/endogenous gene eigenvector; First we considered the hepatic endogenous gene eigenvector u_1 coupled with the sample eigenvector v_1 in Fig 2C. In this eigenvector, the values of u_1 roughly corresponded to the gene expressions of the samples at the bottom end in Fig 2C such as H17L-W18-Gw, H2L-W18-Gw, H12L-W18-Gw, weighted by their values in v_1 due to the SVD structure. Similarly, the negative values of u_1 roughly corresponded to the gene expressions of the samples at the top end in Fig 2C such as R19L-W1, R21L-W1, R17L-W9, weighted by their values in v_1.

We examined what gene subsets were enriched at the two ends of the hepatic endogenous eigenvector. The gene subsets enriched at the bottom end, namely, the mature age end, are shown in Table S2. An obvious group was the immune system including the complement and coagulation cascades, antigen processing and presentation of exogenous peptide antigen via MHC class II, and MHC class I protein complex. A related group was the drug metabolism such as cytochrome P450. Respiration was enhanced along this direction too. Interestingly, the valine, leucine and isoleucine degradation was up-regulated.

Table S2: In the sorted loadings of hepatic first/endogenous gene eigenvector u_1 coupled with v_1 in Fig 2C, gene subsets enriched at the bottom end, namely, the mature age end.

	Gene Subset	Source	P-Value	Bonferroni Correction
**Immune System**	Complement And Coagulation Cascades	KEGG	5.48E-07	1.27E-04
	Antigen Processing And Presentation	Biological Process	3.33E-06	3.32E-03
	Antigen Processing And Presentation Of Exogenous Peptide Antigen Via MHC Class II	Biological Process	3.32E-05	3.32E-02
	MHC Class I Protein Complex	Cellular Component	2.95E-05	2.95E-05
	Autoimmune Thyroid Disease	KEGG	1.11E-05	2.57E-03
**Metabolism**	Valine, Leucine And Isoleucine Degradation	KEGG	9.84E-05	2.27E-02
	Branched-chain amino acid catabolism	Reactome	3.37E-03	
**Drug Metabolism**	Metabolism Of Xenobiotics By Cytochrome P450	KEGG	2.66E-07	6.15E-05
	Drug Metabolism - Cytochrome P450	KEGG	3.40E-06	7.86E-04
	Phase II Conjugation	Reactome	4.22E-05	4.17E-02
	Glutathione Transferase Activity	Molecular Function	9.56E-08	3.23E-05
	Biological Oxidations	Reactome	3.55E-05	3.51E-02

**Respiration**	Oxidative Phosphorylation	KEGG	1.45E-05	3.34E-03
	Respiratory Electron Transport, ATP Synthesis By Chemiosmotic Coupling, And Heat Production By Uncoupling Proteins.	Reactome	9.43E-10	9.31E-07
	Respiratory Chain	Cellular Component	7.20E-08	1.37E-05

The gene subsets enriched at the top end, namely, the young age end, are shown in Table S3. These groups included translation, cell cycle, and apoptosis. The two most significant signaling pathways were the ones of Wnt and Notch.

Table S3: In the sorted loadings of hepatic first/endogenous gene eigenvector $\mathbf{u}_1$ coupled with $\mathbf{v}_1$ in Fig 2C, gene subsets enriched at the top end, namely, the young age end.

	**Gene Subset**	**Source**	**P-Value**	**Bonferroni Correction**
**RNA processing and translation**	RNA Transport	KEGG	2.90e-10	6.69e-08
	Spliceosome	KEGG	3.40e-09	7.85e-07
	Aminoacyl-tRNA Biosynthesis	KEGG	0.000114	2.63e-02
	Aminoacyl-tRNA Ligase Activity	Molecular Function	3.05e-06	
	Translation Initiation Factor Activity	Molecular Function	8.88e-07	
	Ribosome Biogenesis In Eukaryotes	KEGG	6.35e-05	1.47e-02

**Protein**	Protein Processing In Endoplasmic Reticulum	KEGG	1.53e-07	3.54e-05
	Proteasome	KEGG	1.98e-07	4.57e-05
**Cell Cycle**	Regulation Of Mitotic Cell Cycle	Reactome	3.87e-07	3.82e-04
	Cell Cycle Checkpoints	Reactome	4.82e-05	4.75e-02
	Activation Of Apc/C And Apc/C:Cdc20 Mediated Degradation Of Mitotic Proteins	Reactome	2.28e-07	2.25e-04
	Cajal Body	Cellular Component	2.47e-05	0.004722
**Signaling**	Signaling By Wnt	Reactome	9.77e-07	9.65e-04
	Wnt Signaling Pathway	KEGG	0.003873	
	Notch Signaling Pathway	KEGG	0.017899	
**Apoptosis**	Regulation Of Apoptosis	Reactome	2.20e-06	2.17e-03
	Stabilization Of P53	Reactome	9.59e-07	9.47e-04

Hepatic second/HFD gene eigenvector; In the same way, we analyzed the hepatic HFD gene eigenvector u_2 coupled with v_2 as shown in Fig 2F. The gene subsets enriched at the bottom end, namely, the HFD end, are shown in Table S4. We organized them into several groups. The group of fat metabolism included fatty acid, triacylglycerol, and ketone body metabolism, regulation of lipid metabolism by peroxisome proliferator-activated receptor alpha (PPARα), and long-chain fatty acid metabolic process. A closely related subset – the transcriptional regulation of white adipocyte differentiation – was also up-regulated. Structurally, the extracellular matrix related pathways were up-regulated. The activities of ATP-binding cassette transporters, which involve in the transmembrane movement of substances including

metabolic products, lipids and sterols, and drugs, were up-regulated too. Interestingly, the insulin signaling pathway was somewhat enriched at the HFD end.

Table S4: In the sorted loadings of hepatic second/HFD gene eigenvector $\mathbf{u}_2$ coupled with $\mathbf{v}_2$ in Fig 2F, gene subsets enriched at the bottom end, namely, the HFD end.

	**Gene Subset**	**Source**	**P-Value**	**Bonferroni Correction**
**Adipocyte And Fat Metabolism**	Transcriptional Regulation Of White Adipocyte Differentiation	Reactome	0.000343	
	Regulation Of Lipid Metabolism By Peroxisome Proliferator-Activated Receptor Alpha (PPARalpha)	Reactome	0.000344	
	Fatty Acid, Triacylglycerol, And Ketone Body Metabolism	Reactome	0.000351	
	Arrhythmogenic Right Ventricular Cardiomyopathy (ARVC)	KEGG	0.000195	4.50E-02
	Long-Chain Fatty Acid Metabolic Process	Biological Process	0.000322	

**Signaling Pathway**	Jak-STAT Signaling Pathway	KEGG	0.000155	3.58E-02
	Signaling By NOTCH	Reactome	0.000775	
	Pre-NOTCH Transcription And Translation	Reactome	7.03E-06	0.006896
	YAP1- And WWTR1 (TAZ)-Stimulated Gene Expression	Reactome	5.36E-06	0.005258
	TGF-Beta Signaling Pathway	KEGG	0.006464	
	Wnt Signaling Pathway	KEGG	0.009789	
**ATP**	ABC Transporters	KEGG	7.08E-06	1.64E-03
	ATPase Activity, Coupled To Transmembrane Movement Of Substances	Molecular Function	0.000113	0.038148
**Structure**	ECM-Receptor Interaction	KEGG	0.000226	5.21E-02
	Extracellular Matrix Structural Constituent	Reactome	9.69E-05	0.032648
	Beta-Catenin Binding	Molecular Function	1.06E-05	0.003557
**Insulin Signaling Pathway**	PI3K Cascade	Reactome	0.000187	
	PKB-Mediated Events	Reactome	0.000236	
	IRS-Mediated Signaling	Reactome	0.000618	
	mTor Signaling Pathway	KEGG	0.002121	

The gene subsets enriched at the RC end, are shown in Table S5. Equivalently, the

down-enriched functional groups at the HFD end included respiration and translation. Regarding the redox activities, “NADH dehydrogenase (ubiquinone) activity” and “hydrogen ion transmembrane transporter activity” were down-regulated in the HFD group. Strikingly, steroid biosynthesis, and cholesterol biosynthesis were down-regulated overwhelmingly at the HFD end.

Table S5: The sorted loadings of hepatic second/HFD gene-eigenvector u_2 coupled with v_2 in Fig 2F, gene subsets up-enriched at the top end, namely, the RC end.

	Gene Subset	Source	P-Value	Bonferroni Correction
**Respiration**	Oxidative Phosphorylation	KEGG	5.46E-10	1.26E-07
	Mitochondrial Respiratory Chain Complex I	Cellular Component	2.06E-06	3.93E-04
	Mitochondrial Proton-Transporting ATP Synthase Complex	Cellular Component	4.80E-05	9.17E-03
	ATP Synthesis Coupled Proton Transport	Biological Process	1.23E-05	0.012337
**Steroid Biosynthesis**	Steroid Biosynthesis	KEGG	1.94E-05	0.004476
	Steroid Hormone Biosynthesis	KEGG	0.010399	
	Cholesterol Biosynthesis	Reactome	4.23E-10	4.15E-07
	Sterol Biosynthetic Process	Cellular Component	1.50E-09	1.50E-06
**Translation**	Translation	Reactome	5.55E-17	5.44E-14
	Ribosome Biogenesis In Eukaryotes		0.000589	
	Cytosolic Small Ribosomal Subunit	Cellular Component	5.91E-07	1.13E-04
	Ribosome	KEGG	9.98E-14	2.31E-11
**Redox**	NADH	Molecular	0.001447	

	Dehydrogenase (Ubiquinone) Activity	Function		
	Hydrogen Ion Transmembrane Transporter Activity	Molecular Function	0.001055	

Adipose first gene eigenvector; Next we analyzed the first adipose gene eigenvector u_1 coupled with v_1 in Fig 3A. The gene subsets enriched at the bottom end, namely, the HFD and mature age end, are shown in Table S6. Most enriched gene subsets in the up-regulated genes were related to inflammation. This can be seen from several views. First, gene subsets of phagocytosis defined in KEGG, GO-biological process, and REACTOME, were all up-regulated with great statistical significance. The GO biological process "antigen processing and presentation of exogenous peptide antigen via MHC class II" was significantly up-regulated. The KEGG signaling pathways relating to the immune system such as chemokine signaling pathway, B cell receptor signaling pathway, the cytokine-cytokine receptor interaction, and toll-like receptor signaling pathway were all up-enriched. Regarding metabolisms, steroid and cholesterol biosynthesis were enriched at the HFD end. Interestingly, insulin receptor recycling was enriched too.

Table S6: In the sorted loadings of adipose first gene-eigenvector u_1 coupled with v_1 in Fig 3A, gene subsets enriched at the bottom end, namely, the HFD end.

	**Gene Subset**	**Source**	**P-Value**	**Bonferroni Correction**
**Phagocytosis**	Phagosome	KEGG	3.24E-13	7.46E-11
	Positive Regulation Of Phagocytosis	Biological Process	2.42E-05	2.38E-02
	Phagosomal Maturation (Early Endosomal Stage)	Reactome	5.52E-07	0.000542
	Phagocytosis, Engulfment	Biological Process	0.000803	
**Immune Response**	Antigen Processing And Presentation Of	Biological Process	2.09E-07	2.06E-04

	Exogenous Peptide Antigen Via MHC Class II			
	MHC Class I Protein Complex	Cellular Component	0.003758	
	B Cell Receptor Signaling Pathway	KEGG	1.09E-05	0.002496
	Intestinal Immune Network For Iga Production	KEGG	0.000342711	
	Leukocyte Cell-Cell Adhesion	Biological Process	2.19E-05	
**Signaling Pathway**	Chemokine Signaling Pathway	KEGG	2.77E-08	6.37E-06
	Toll-Like Receptor Signaling Pathway	KEGG	8.95E-05	0.020578
	Cytokine-Cytokine Receptor Interaction	KEGG	0.000107986	
	Chemokine Receptors Bind Chemokines	Reactome	1.72E-05	0.016947
**Secretion**	Positive Regulation Of Tumor Necrosis Factor Production	Biological Process	0.001892	
	Positive Regulation Of Interferon-Gamma Production	Biological Process	0.001435	
	Positive Regulation Of Cytokine Secretion	Biological Process	0.000173	

	Positive Regulation Of Interleukin-6 Production	Biological Process	0.010029	
	Negative Regulation Of Interleukin-6 Production	Biological Process	0.022957	
	Positive Regulation Of Interleukin-10 Production	Biological Process	0.00374	
	Positive Regulation Of Interleukin-12 Production	Biological Process	0.019111	
**Biosynthesis**	Steroid Biosynthesis	KEGG	0.000902576	
	Positive Regulation Of Cytokine Secretion	Biological Process	0.000173	
	Leukotriene Biosynthetic Process	Biological Process	0.000237	
	Cholesterol Biosynthesis	Reactome	0.001748	
**ROS**	Positive Regulation Of Nitric Oxide Biosynthetic Process	Biological Process	0.000725	
	Superoxide Metabolic Process	Biological Process	0.001197	
	Positive Regulation Of Reactive Oxygen Species Metabolic Process	Biological Process	0.008742	
**Diabetes**	Insulin Receptor Recycling	Reactome	0.002983	

We then examined the gene subsets up-enriched gene subsets at the top end or down-enriched at the HFD end of the gene-eigenvector for the adipose tissue, see Table S7. It is not a surprise that the insulin signaling pathway was down-regulated at the HFD or the mature age end, and this was an indicator of insulin resistance. Along the same direction, downstream of glucose intake, an REACTOME pathway, "carbohydrate-responsive element-binding protein (ChREBP) activation of metabolic gene" was down-regulated. Furthermore, other metabolisms such as fatty acid, triacylglycerol, and ketone body metabolism, regulation of lipid metabolism by PPAR, and branched-chain amino acid (BCAA) catabolism, were significantly down-regulated. From the angle of the cell differentiation, the transcriptional regulation of white adipocyte differentiation was down-regulated. Nuclear receptors belong to an important class of transcription factors that are responsible for sensing steroid and thyroid hormones and certain other molecules. It turns out their transcription pathway was down-regulated along with the insulin sensitivity. The steroid hormone receptor activity reduced correspondingly. The regulation of circadian rhythm was modified too.

Table S7: In the sorted loadings of adipose first gene eigenvector u_1 coupled with v_1 in Fig 3A, gene subsets enriched at the top end, namely, the RC end.

	**Gene Subset**	**Source**	**P-Value**	**Bonferroni Correction**
**Insulin Signaling**	Insulin Signaling Pathway	KEGG	0.006861	
	Insulin-Like Growth Factor Receptor Binding	Molecular Function	0.000298	
**BCAA Degradation**	Valine, Leucine And Isoleucine Degradation	KEGG	0.001988	
	Branched-Chain Amino Acid Catabolism	Reactome	9.54E-05	
**Receptor**	Nuclear Receptor Transcription Pathway	Reactome	0.000306	
	Ligand-Dependent Nuclear Receptor Activity	Molecular Function	0.00044	
	Steroid Hormone Receptor Activity	Molecular Function	8.01E-05	2.68E-02

	Steroid Hormone Mediated Signaling Pathway	Biological Pathway	2.50E-05	2.49E-02
**Metabolism**	Fatty Acid, Triacylglycerol, And Ketone Body Metabolism	Reactome	9.64E-08	9.47E-05
	Regulation Of Lipid Metabolism By Peroxisome Proliferator-Activated Receptor Alpha (Pparalpha)	Reactome	5.10E-05	5.01E-02
	Very Long-Chain Fatty Acid Metabolic Process	Biological Process	0.0095	
	Chrebp Activates Metabolic Gene Expression	Reactome	0.000177	
	Metabolism Of Water-Soluble Vitamins And Cofactors	Reactome	0.00108	
	Carbonate Dehydratase Activity	Molecular Functions	5.45E-05	1.82E-02
	Nitrogen Metabolism	KEGG	0.00017	0.040
**Respiration**	The Citric Acid (TCA) Cycle And Respiratory Electron Transport	Reactome	0.0018	
	Mitochondrial Membrane	Cellular Component	0.00066	
**Circadian Rhythm**	Circadian Repression Of Expression By Rev-Erba	Reactome	0.00080	
	Rora Activates Circadian Expression	Reactome	0.0013	
	Bmal1:Clock/Npas2 Activates Circadian Expression	Reactome	0.0032	
**Peroxisome**	Peroxisome	KEGG	0.023	
	Peroxisomal Membrane	Cellular Component	0.0034	

**Adipocyte Differentiation**	Transcriptional Regulation Of White Adipocyte Differentiation	Reactome	0.0024	
**Blood Vessel**	Vasculogenesis	Biological Process	0.0033	
	Blood Vessel Development	Biological Process	0.0089	
	Regulation Of Blood Vessel Size	Biological Process	0.007494	
	Positive Regulation Of Endothelial Cell Migration	Biological Process	0.008889	
	Epithelial Cell Differentiation	Biological Process	0.0022	
	VEGF Binds To VEGFR Leading To Receptor Dimerization	Reactome	0.058308	

Pancreatic first/HFD gene eigenvector; The enriched gene subsets at the two end of the pancreatic first or HFD gene eigenvector u_1 coupled with v_1, as shown in Fig 3B, were examined next. The up-regulated ones at the H7P-W9 and H35P-W9 end are shown in Table S8. Similar to the first hepatic gene eigenvector, the immune and drug metabolism was up-regulated together with the KEGG Chemokine signaling pathway, Jak-STAT signaling pathway, PPAR signaling pathway, cellular response to interferon-beta *et al*. The metabolism of fat and lipid was also modified, as evidenced in an REACTOME pathway: PPAR activated gene expression, fatty acid, triacylglycerol, and ketone body metabolism, and metabolism of lipids and lipoproteins. Notably, the up-regulation also included positive regulation of angiogenesis, vasculogenesis, palate development, etc. Along the structural dimension, caveola and stress fiber showed significant enrichment.

Table S8: In the sorted loadings of the pancreatic first gene-eigenvector u_1 coupled with v_1 in Figure 3B, gene subsets enriched at the bottom end, namely, H7P-W9 and H35P-W9 end.

	**Gene Subset**	**Source**	**P-Value**	**Bonferroni Correction**
**Immune And Drug Metabolism**	Complement And Coagulation Cascades	KEGG	9.82E-11	2.26E-08

	Innate Immune System	Reactome	2.24E-0	2.21E-03
	MHC Class I Protein Complex	Cellular Component	4.77E-06	9.25E-04
	Antigen Processing And Presentation	Biological Process	3.69E-05	3.68E-02
	Intestinal Immune Network For Iga Production	KEGG	0.000109	2.51E-02
	Positive Regulation Of B Cell Proliferation	Biological Process	0.000282	
	Positive Regulation Of T Cell Differentiation	Biological Process	0.001602	
	Metabolism Of Xenobiotics By Cytochrome P450	KEGG	0.000103	2.36E-02
	Phagosome	KEGG	0.000868	
	Leishmaniasis	KEGG	1.01E-06	2.33E-04
	Biological Oxidations	Reactome	1.33E-05	1.31E-02
**Signaling Pathway**	Cytokine-Cytokine Receptor Interaction	KEGG	3.46E-09	7.95E-07
	Chemokine Signaling Pathway	KEGG	5.59E-07	1.29E-04
	Jak-STAT Signaling Pathway	KEGG	8.11E-06	1.86E-03
	Cellular Response To Interferon-Beta	Biological Process	8.14E-06	8.11E-03
	PPAR Signaling Pathway	KEGG	0.000145	3.33E-02
	Receptor Complex	Cellular Component	0.000252	4.89E-02
**Fat and Lipid Metabolisms**	PPARA Activates Gene Expression	Reactome	0.000461	
	Fatty Acid, Triacylglycerol, And Ketone Body Metabolism	Reactome	0.000657	
	Metabolism Of Lipids And Lipoproteins	Reactome	3.66E-07	3.61E-04
	High-Density Lipoprotein Particle	Cellular Component	9.66E-05	1.87E-02

**Growth**	Positive Regulation Of Angiogenesis	Biological Process	6.62E-06	6.59E-03
	Vasculogenesis	Biological Process	4.81E-05	4.79E-02
	Regulation Of Cell Growth	Biological Process	8.46E-06	8.42E-03
	Brown Fat Cell Differentiation	Biological Process	0.000162	
	Growth Factor Binding	Molecular Function	7.94E-07	2.64E-04
	Insulin-Like Growth Factor Binding	Molecular Function	8.50E-06	2.83E-03
	Signaling by VEGF, VEGF Ligand-Receptor Interactions	Reactome	0.010287	
**Structure**	Caveola	Cellular Component	2.61E-07	5.07E-05
	Stress Fiber	Cellular Component	7.17E-05	1.39E-02

The gene subsets up-enriched at the RC end or down-enriched at the H7P-W9 and H35P-W9 end for the pancreatic HFD eigenvector are shown in Table S9. Apoptosis was down-regulated significantly along with protein synthesis and proteolysis. Respiration was reduced. Regarding redox, GO molecular function gene subset of NADH dehydrogenase (ubiquinone) activity was reduced. Interestingly, the KEGG pathway – Maturity onset diabetes of the young (MOD), and insulin receptor recycling that are related to diabetes were also down-regulated at the end of H7P-W9 and H35P-W9.

Table S9: In the sorted loadings of the pancreatic first gene-eigenvector $\mathbf{u_1}$ coupled with $\mathbf{v_1}$ in Figure 3B, gene subsets up-enriched at the top or RC end, namely, gene subsets down-enriched at the H7P-W9 and H35P-W9 end.

	**Gene Subset**	**Source**	**P-Value**	**Bonferroni Correction**
**Apoptosis**	Regulation Of Apoptosis	Reactome	1.47E-11	1.45E-08
	P53-Independent G1/S DNA Damage Checkpoint	Reactome	2.36E-12	2.33E-09

	P53-Dependent G1/S DNA Damage Checkpoint	Reactome	2.68E-10	2.65E-07
	Activation Of Chaperones By IRE1alpha	Reactome	1.68E-05	0.016587
	Activation Of Chaperone Genes By XBP1(S)	Reactome	3.64E-05	0.035957
**Redox**	NADH Dehydrogenase (Ubiquinone) Activity	Molecular Function	3.00E-05	0.009974
	Protein Disulfide Oxidoreductase Activity	Molecular Function	0.000146	0.048556
**Protein**	Translational Initiation	Biological Process	4.66E-13	4.65E-10
	Ribosome Biogenesis In Eukaryotes	KEGG	4.93E-05	0.011336
	Protein Processing In Endoplasmic Reticulum	KEGG	5.15E-13	1.18E-10
**Proteasome**	Proteasome	KEGG	3.38E-12	7.78E-10
	Proteolysis Involved In Cellular Protein Catabolic Process	Biological Process	2.13E-07	2.13E-04
**Respiration**	Oxidative Phosphorylation	KEGG	2.45E-14	5.63E-12
	Respiratory Electron Transport, ATP Synthesis By Chemiosmotic Coupling, And Heat Production By Uncoupling	Reactome	1.04E-13	1.02E-10

	Proteins.			
	Mitochondrial Respiratory Chain Complex I	Cellular Component	2.72E-08	5.27E-06
**Diabetes**	Maturity Onset Diabetes Of The Young	KEGG	0.001711	

Pancreatic second/development gene eigenvector; Earlier we postulated that the second pancreatic sample eigen-component primarily corresponded to the pancreas development. This can further be verified by the gene eigenvector u_2 coupled with v_2 in Fig 3C. The enriched gene subsets at the mature age end are shown in Table S10. An REACTOME pathway – Regulation of gene expression in beta cells, and a KEGG pathway – Pancreatic secretion, were up-regulated significantly even after Bonferroni correction. A related REACTOME gene subset – MOD pathway was also up-enriched. In terms of regulation, the REACTOME gene subset, the steroid hormone receptor activity, were enriched. Another up-enriched group was translational activities including the KEGG ribosome pathways.

Table S10: In the sorted loadings of the pancreatic second/development gene eigenvector u_2 coupled with v_2 in Figure 3C, gene subsets enriched at bottom or the mature age end.

	**Gene Subset**	**Source**	**P-Value**	**Bonferroni Correction**
**Translation**	Ribosome	KEGG	4.68E-05	1.08E-02
	Eukaryotic Translation Elongation	Reactome	9.51E-06	9.39E-03
	Eukaryotic Translation Termination	Reactome	3.14E-05	3.10E-02
**Steroid Hormone**	Steroid Hormone Receptor Activity	Reactome	1.68E-05	0.016587
	Steroid Hormone Mediated Signaling Pathway	Biological Process	0.000275	
	Sterol Biosynthetic Process	Biological Process	0.001788	
	Cholesterol Biosynthetic Process	Biological Process	0.001955	
	Steroid Hormone	Molecular	0.00102	

	Receptor Activity	Function		
**Nuclear Receptor**	Ligand-Dependent Nuclear Receptor Activity	Molecular Function	0.002363	
	Nuclear Receptor Transcription Pathway	Reactome	0.00294	
**Pancreas**	Regulation of Gene Expression In Beta Cells	Reactome	4.66E-13	4.65E-10
	Pancreatic Secretion	KEGG	2.13E-07	2.13E-04
**Diabetes**	Maturity Onset Diabetes of The Young	KEGG	0.001711	
	Diabetes Pathways	Reactome	1.54E-05	0.015216
	Type 2 Diabetes Mellitus	KEGG	0.010658	
**Insulin Signaling**	Phosphatidylinositol 3-Kinase Complex	Cellular Component	0.005672	
	PI3K Events in ERBB2 Signaling	Reactome	1.04E-13	
	Erbb Signaling Pathway	KEGG	0.002143	
	Inositol or Phosphatidylinositol Kinase Activity	Molecular Function	0.002161	
	Positive Regulation of Glucose Import	Biological Process	0.006365	
	Insulin Receptor Signaling Pathway	Biological Process	0.004026	
**Histone**	Histone H3 Acetylation	Biological Process	0.00123	
**Metabolisms**	Drug Metabolism - Cytochrome P450	KEGG	0.001356	
	Linoleic Acid Metabolism	KEGG	0.00089	
	Valine, Leucine and Isoleucine Degradation	KEGG	0.004042	
	Branched-Chain Amino Acid Catabolism	Reactome	0.008545	

The gene subsets down-enriched at the mature age end or up-enriched at the young age end of the pancreatic second/development gene eigenvector u_2 were shown in Table S11. The immune system group included a REACTOME pathway – Antigen processing – Cross presentation. The Platelet group included REACTOME pathways – Platelet degranulation and Response to elevated platelet cytosolic Ca2+. Another REACTOME pathway – Signaling by Wnt – was down-enriched at the mature age end. Like the first gene eigenvector u_1, proteasome and apoptosis were down-enriched at the mature age end.

Table S11: In the sorted loadings of the pancreatic second gene-eigenvector u_2 coupled with v_2 in Figure 3C, gene subsets enriched at the top end, namely, the young age end.

	**Gene Subset**	**Source**	**P-Value**	**Bonferroni Correction**
**Proteasome**	Proteasome	KEGG	1.04E-08	2.39E-06
	Proteasome Core Complex	Cellular Component	1.07E-05	
**Signaling**	Signaling By Wnt	Reactome	1.23E-08	1.22E-05
**Apoptosis**	Regulation Of Apoptosis	Reactome	4.60E-07	4.54E-04
	P53-Independent DNA Damage Response	Reactome	6.50E-08	6.41E-05
	P53-Dependent G1 DNA Damage Response	Reactome	1.77E-06	1.74E-03
**Immune**	Antigen Processing-Cross Presentation	Reactome	1.71E-10	1.68E-07
	Activation Of NF-Kappab In B Cells	Reactome	1.56E-06	1.54E-03
**Platelet**	Platelet Degranulation	Reactome	1.61E-06	1.59E-03
	Response To Elevated Platelet Cytosolic Ca2+	Reactome	5.71E-06	5.63E-03

Different patterns of inflammation- and macrophage-related gene expression in adipose and pancreatic tissues. HFD can induce a chronic low-grade inflammatory state associated with obesity [7]. In the process of such inflammation development, macrophage infiltration plays an important role [8]. Macrophage is one kind of phagocytes involving phagocytosis. At the HFD end of the adipose first gene eigenvector, we observed the significant up-enrichment of phagocytosis activities such as the KEGG Phagosome pathway and the GO biological process of positive regulation of phagocytosis (Table S6). We further checked the loadings and ranks of the genes as reported by Chen's group [9] that are either macrophage-specific or possibly related to the macrophage recruitment in the adipose tissue (Table S12). They included: *Adam8* (a disintegrin and metallopeptidase domain 8), *MCP-1* (Ccl2, chemokine ligand 2), *Ccr2* (chemokine receptor 2), *F4/80* (a classical macrophage-restricted surface glycoprotein), *CD68* (CD68 antigen or macrosialin), *Mpeg1* (macrophage expressed gene 1), and *Msr1* (macrophage scavenger receptor 1). All these genes ranked high in the loadings of the adipose first gene eigenvector.

Moreover, the significance levels of the positive regulation of productions for pro-inflammatory cytokines including tumor necrosis factor (*TNF*), interferon-gamma, regulation of interleukin 6 (*IL-6*), and productions of several other factors were listed in Table S6. The toll receptor cascades, which have been proposed to mediate the inflammation process, were all significantly up-regulated along with the pro-

inflammatory factors.

More details of the phagocytosis activities were obtained by pairwise sample comparisons. That is, for a specific pathway, we compared the expression profiles of a pair of samples from different diet groups at a certain age/time by the Wilcoxon rank test. The fractions of up-regulated pairs were shown in Table S13, in which the significance level was taken to be 0.05. It is not surprising that the list included the KEGG pathways Leishmaniasis and Tuberculosis because these diseases are closely related to macrophage. Other than phagocytosis, Antigen processing and presentation (KEGG pathway) and Antigen processing and presentation of exogenous peptide antigen via MHC class II (GO biological process) were also significantly up-enriched. Thus the landscape of the immune system of the adipose tissue was indeed modified by the HFD stress.

For the pancreas, the up-enriched gene subsets at the HFD end along the first/HFD gene eigenvector were listed in Table S8. Similar to what were up-enriched at the HFD end in the adipose eigenvector, they included phagocytosis activities such as Phagosome (KEGG) and Leishmaniasis (KEGG), Chemokine Signaling Pathway, Antigen Processing and Presentation (GO biological process). The enrichment mainly came from samples of week 9. The pairwise sample comparisons of the RC and HFD groups at week 9 were shown in the last column of Table S13. However, the chemoattractant *MCP-1, F4/80,* and *Adam8* did not contribute significant loadings in

pancreatic HFD gene eigenvector. Instead, the loading of *Mmd* (the monocyte to macrophage differentiation-associated gene) ranked 28.

Table S12: The macrophage-related inflammation genes, and their loadings and ranks in the adipose first gene eigenvector.

Gene symbols	Loading value	Ranks	Gene
Adam8	0.061123	10	a disintegrin and metallopeptidase domain 8
Mpeg1	0.049602	21	macrophage expressed gene 1
Mcp-1/Ccl2	0.043217	38	chemokine (C-C motif) ligand 2
Ccr2	0.029555	144	chemokine (C-C motif) receptor 2
Emr1 F4/80	0.031888	110	EGF-like module containing, mucin-like, hormone receptor-like sequence 1
CD14	0.022857	280	CD14 antigen
CD68	0.038717	59	CD68 antigen
Msr1	0.035645	82	macrophage scavenger receptor 1
Csf2ra	0.032671	99	colony stimulating factor 2 receptor, alpha, low-affinity (granulocyte-macrophage)
Mif	-0.00053	15436	macrophage migration inhibitory factor

Table S13: Pairwise sample comparisons for the macrophage-related pathways, inflammation pathways, and their signaling pathways. That is, at a certain time point, we compare a pair of samples from different groups, using the expressions from one specific pathway by the Wilcoxon rank test. The fractions of up-regulated pairs are shown, in which the significance level is taken to be 0.05.

Gene Subsets	Source	**HFD-W9** vs **RC-W9, adipose**	**HFD-W18-Gw** vs **RC-W18, adipose**	**HFD-W18-Gw** vs **HFD-W18-Gb adipose**	**HFD-W9** vs **RC-W9, pancreas**
Phagosome	KEGG	9/9	6/6	4/6	8/9
Leishmaniasis	KEGG	9/9	6/6	5/6	9/9
Tuberculosis	KEGG	9/9	6/6	4/6	8/9
Positive Regulation Of	Biological process	9/9	6/6		

Tumor Necrosis Factor Production					
I-Kappab Kinase/NF-Kappab Cascade	Biological process	6/9	5/6		
Toll-Like Receptor Signaling Pathway	KEGG	8/9	6/6	6/6	
Toll Receptor Cascades	Reactome	6/9	6/6	4/6	8/9
Chemokine Signaling Pathway	KEGG	8/9	6/6	5/6	9/9
Antigen Processing And Presentation	KEGG	9/9	6/6	5/6	8/9
Antigen Processing And Presentation Of Exogenous Peptide Antigen Via MHC Class II	Biological process	8/9	5/6	4/6	9/9
ECM-Receptor Interaction	KEGG	8/9			7/9

Profound down-regulation of cholesterol/steroid biogenesis in the hepatic HFD gene eigenvector. When we checked the hepatic HFD gene eigenvector in details, it turned out that almost all the genes of the cholesterol biogenesis pathway were at the very end of down-regulation. They included the top six genes: *Sqle* (squalene epoxidase), *Idi1* (isopen-tenyl-diphosphate delta isomerase), *Fdps* (farnesyl diphosphate synthetase), *Cyp51* (cytochrome P450, family 51), *Sc4mol* (sterol-C4-methyl oxidase-like), and *Nsdhl* (NAD(P) dependent steroid dehydrogenase-like). In the top-35 list, eight other genes were in the cholesterol biogenesis pathway: *Pmvk* (phosphomevalonate kinase), *Hmgcs1* (3-hydroxy-3-methylglutaryl-Coenzyme A synthase 1), *Dhcr7* (7-dehydrocholesterol reductase), *Lss* (lanosterol synthase), *Mvd* (mevalonate decarboxylase), *Fdft1* (farnesyl diphosphate farnesyl transferase 1), *Apoe* (apolipoprotein E), and *Sc5d* (sterol-C5-desaturase). Fig. S4 and Table S14 showed the loadings of the hepatic HFD gene eigenvector on the KEGG steroid biosynthesis pathway, where down-regulation was marked in blue and up-regulation was marked in red. This pathway is upstream of bile acid, cholesterol, and steroid hormone biosynthesis. Dhcr24 (24-dehydrocholesterol reductase. EC:1.3.1.72), which is up-regulated substantially, is an exception in this KEGG pathway. Next we applied the BASE2.0 method to the hepatic HFD gene eigenvector to detect the enriched regulatory motifs around the gene regulatory regions. The most significantly enriched TRANSFAC motifs included those for Sterol Regulatory Element-Binding Proteins SREBP _Q3 (p-value≈0.001), SREBP1_02 (p-value≈0.0034), SREBP1_Q5 (p-value≈0.0064), and SREBP1_Q6 (p-value≈0.0068, see Fig. S5 for its logo). Their

binding transcription factors are SREBP1A/1C or SREBP2. At the expression level, the loading of *Srebf1* (sterol regulatory element binding transcription factor 1) ranked 20 at the HFD end of the gene eigenvector, while that of Srebf2 (sterol regulatory element binding factor 2) was near zero. SREBP2 controls cholesterol homeostasis by stimulating transcription of sterol-regulated genes [10].

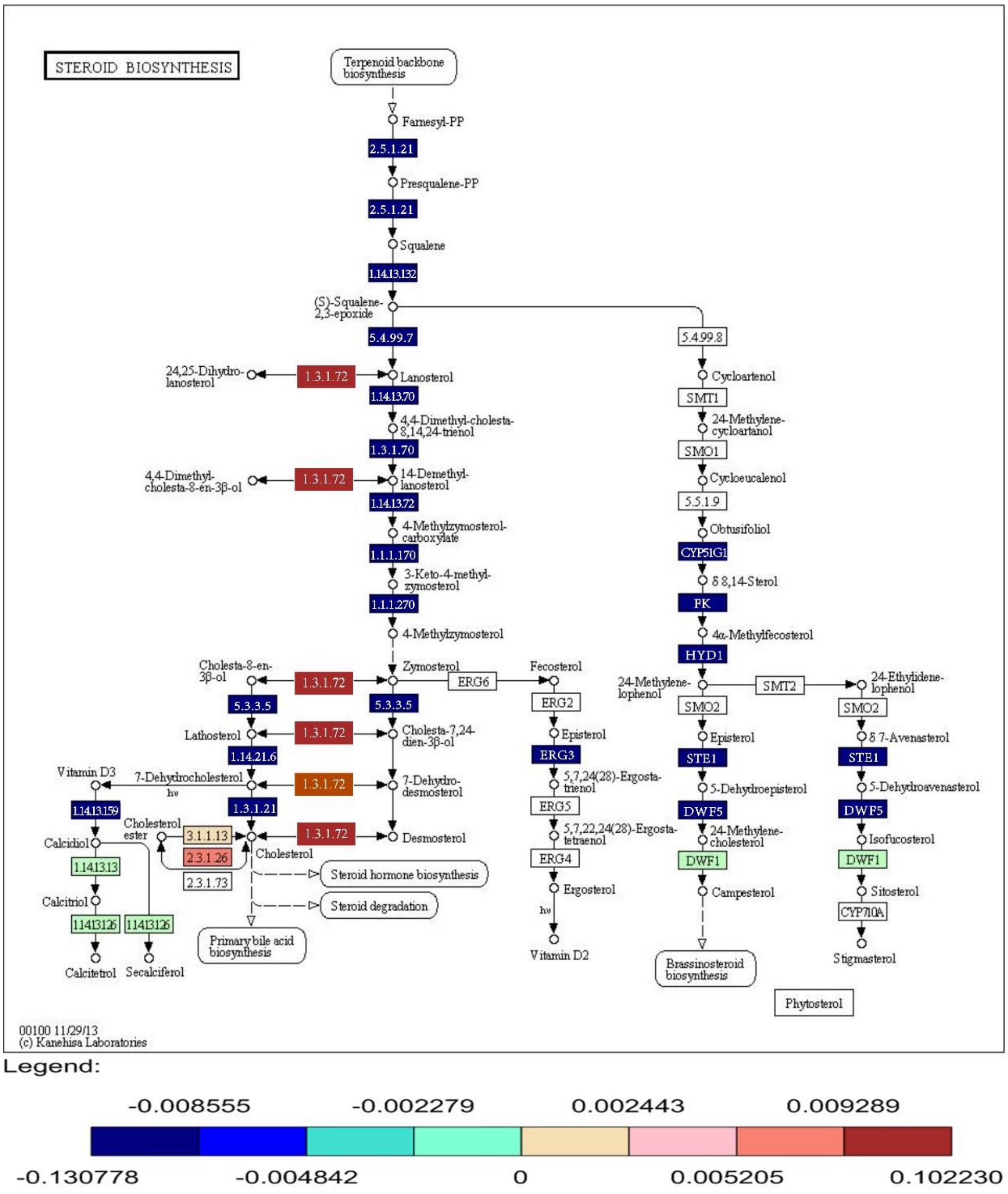

Fig. S4: The loadings of hepatic second/HFD gene eigenvector on the Steroid Biogenesis pathway (KEGG[11]). The down-regulation is marked in blue and up-regulation is marked in red. Some genes are represented by EC numbers [12], whose

corresponding gene symbols can be found in Table S15.

Table S14: The genes in the Steroid Biogenesis pathway (KEGG) shown in Fig. S4 and related pathways. From left to right are their gene symbols, EC numbers, loadings in the hepatic second/HFD gene eigenvector, ranks (negative value means ranks from the negative end), and GenMAPP [13] pathways they belong to.

Symbol	EC	Value	Rank	Pathway (GenMAPP)
Sqle	EC:1.14.99.7	-0.1415	-1	Cholesterol_Biosynthesis
Idi1		-0.1104	-2	Cholesterol_Biosynthesis; Circadian_Exercise
Fdps	EC:2.5.1.1, EC:2.5.1.10	-0.09945	-3	Cholesterol_Biosynthesis
Cyp51	EC:1.14.13.70	-0.08851	-4	Cholesterol_Biosynthesis
Sc4mol	EC:1.14.13.72	-0.08562	-5	Cholesterol_Biosynthesis
Nsdhl	EC:1.1.1.170	-0.08366	-6	Cholesterol_Biosynthesis
Pmvk		-0.0692	-10	Cholesterol_Biosynthesis
Hmgcs1	EC:2.3.3.10, EC:4.1.3.5	-0.05576	-15	Cholesterol_Biosynthesis
DWF5(Dhcr7)	EC:1.3.1.21	-0.05424	-16	Cholesterol_Biosynthesis
FK(Tm7sf2)		-0.05065	-19	
Hmgcr		-0.05003	-20	Cholesterol_Biosynthesis; Statin_Pathway_PharmGKB
Lss	EC:5.4.99.7	-0.04865	-22	Cholesterol_Biosynthesis
Acss2		-0.04774	-23	
Elovl3		-0.04703	-24	
Mvd		-0.04574	-25	Cholesterol_Biosynthesis
Fdft1	EC:2.5.1.21	-0.04551	-26	Cholesterol_Biosynthesis
Apoe		-0.0454	-27	Statin_Pathway_PharmGKB
Sigmar1		-0.04326	-30	
Cdh1		-0.04265	-31	Cell_Cycle_KEGG
EGR3/STE1 (Sc5d)	EC:1.14.21.6	-0.04172	-34	Cholesterol_Biosynthesis
Adipoq		-0.04076	-35	
Hsd17b7	EC:1.1.1.270, EC:1.1.1.62	-0.03579	-45	Steroid_Biosynthesis
Mvk	EC:2.7.1.36	-0.03234	-58	Cholesterol_Biosynthesis
Cyp2r1	EC:1.14.13.15	-0.0323	-59	
HYD1(Ebp)	EC:5.3.3.5	-0.01372	-518	
Cyp27b1	EC:1.14.13.13	0	-8022	
Cyp24a1	EC:1.14.13.126	0	-4941	
Dhcr24	EC:1.3.1.72	0.02807	67	
Soat1	EC:2.3.1.26	0.00943	1449	Statin_Pathway_PharmGKB

Soat2	EC:2.3.1.26	0.02053	192	Smooth_muscle_contraction
Cel	EC:3.1.1.13	0.01084	1145	

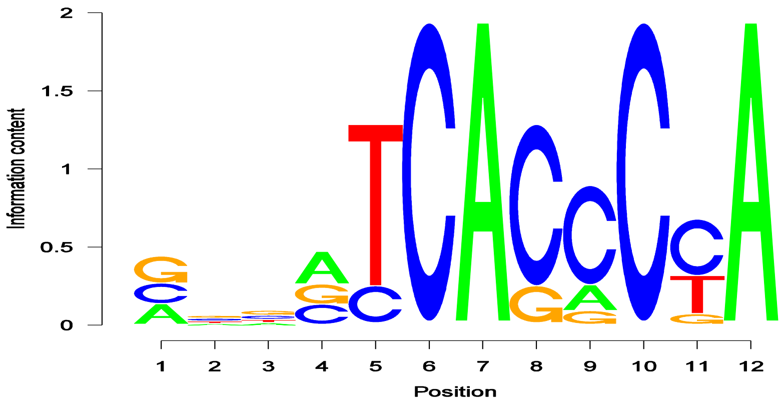

Fig. S5: The logo of the Steroid Response Element, SREBP_Q3 from TRANSFAC.

Diverse expression patterns of BCAA degradation pathway in the adipose, the liver, and the pancreas. In the main text, we showed that the gene eigenvectors displayed different regulation patterns of BCAA degradation across tissues. Next we directly compared the HFD and RC groups for different tissues along the time course of the experiments (Table S15). We observed opposite regulations of BCAA degradation pathway in the adipose and pancreas tissues, namely, down-regulation in the adipose tissue while up-regulation in the pancreas. For the adipose tissue, at week 9, eight out of nine (8/9) pair-wise comparisons were significantly down-regulated for the KEGG pathway, and 9/9 comparisons were so for the REACTOME pathway. The comparison was regarded as significant if the p-value was less than 0.05. At week 18, the down-regulation was significant across the board. Impaired glucose tolerance and insulin resistance as measured by GTT/ITT results were associated with the HFD (Fig. 2A), and thus associated with BCAA degradation in the adipose tissue. This association between GTT and down-regulated BCAA degradation pathway in the adipose tissue was further validated by comparing the samples from the HFD-W18-Gw and the HFD-W18-Gb subgroups. Five out of six (5/6) pairs were significantly down-regulated for the KEGG pathway and 4/6 pairs were significant for the REACTOME pathway. In comparison, most pairwise comparisons between the two diet groups in the pancreas showed up-regulation at week 18: 5/6 for the KEGG pathway and 6/6 for the REACTOME pathway. At week 9, 6/9 pairs showed up-regulation for the KEGG pathway. The results of BCAA degradation in the liver samples were not as impressive as in the adipose and pancreas. At week 18, 6/9 pairs showed up-regulation for the KEGG pathway for the liver samples.

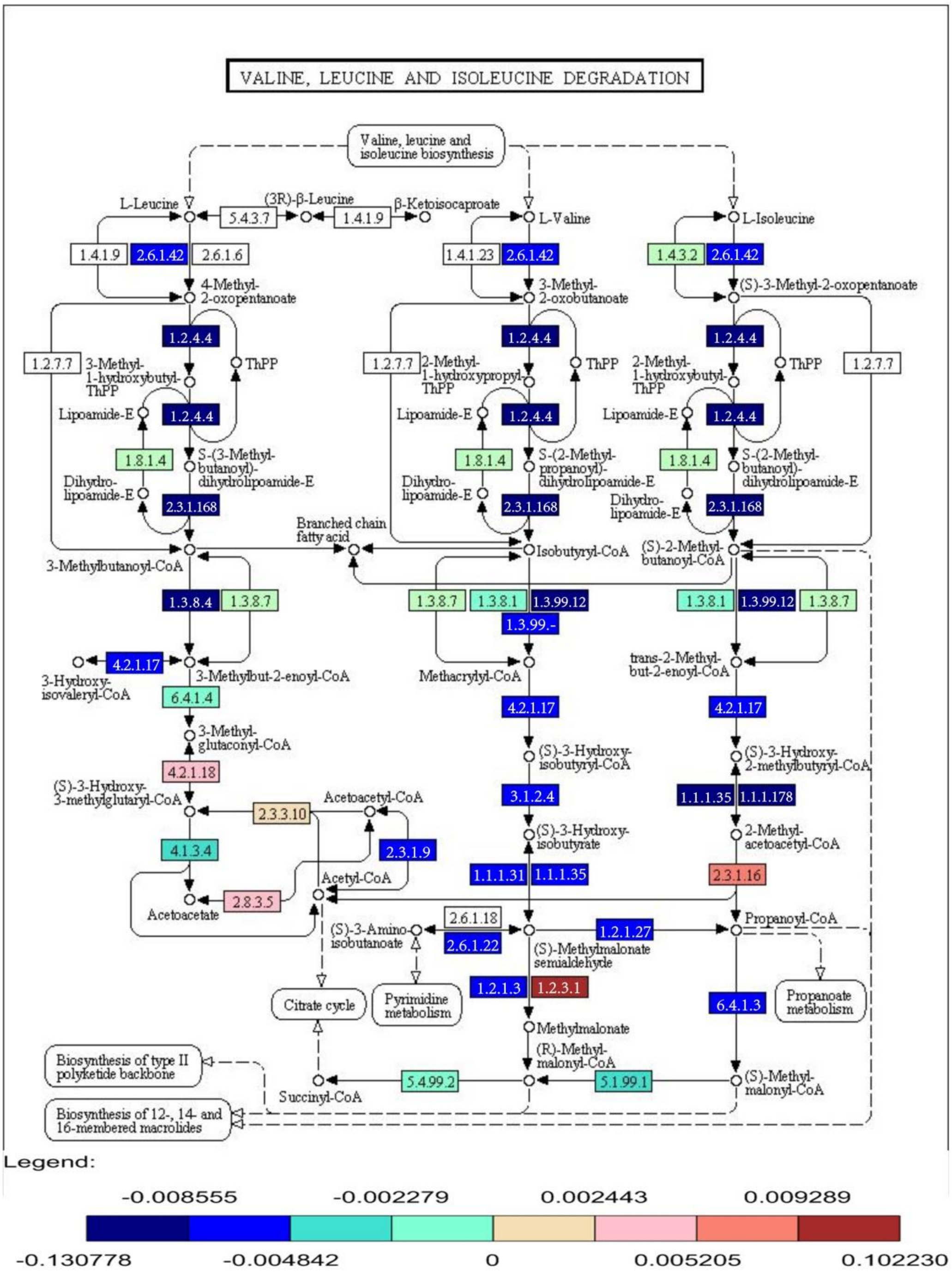

Fig. S6: The loadings of adipose first gene eigenvector on the KEGG BCAA degradation pathway[11]. The down-regulation is marked in blue and up-regulation is marked in red. The key genes Bcat1 and Bcat2 are represented by the node of EC: 2.6.1.42, and the genes Bckdha and Bckdhb are represented by the node of EC: 1.2.4.4.

Table S15: The regulation of BCAA degradation shows opposite directions in the adipose, the pancreas, and the liver. At a certain time point, we compare a pair of samples from different groups, using the expressions from the BCAA degradation pathway by the Wilcoxon rank test. The fractions of up- or down-regulated pairs are shown, in which the comparison is regarded as significant if the p-value is less than 0.05.

	HFD vs RC, Week 9, adipose	HFD vs RC, Week 18, adipose	HFD-W vs HFD-B, Week 18, Adipose	HFD vs RC, Week 9 pancreas	HFD vs RC, Week 18, pancreas	HFD vs RC, Week 18, Liver
**Valine, leucine and isoleucine degradation KEGG**	8/9(down)	9/9(down)	5/6(down)	6/9(up)	5/6(up)	6/9(up)
**Branched-chain amino acid catabolism, reactome**	9/9(down)	9/9(down)	4/6(down)		6/6(up)	

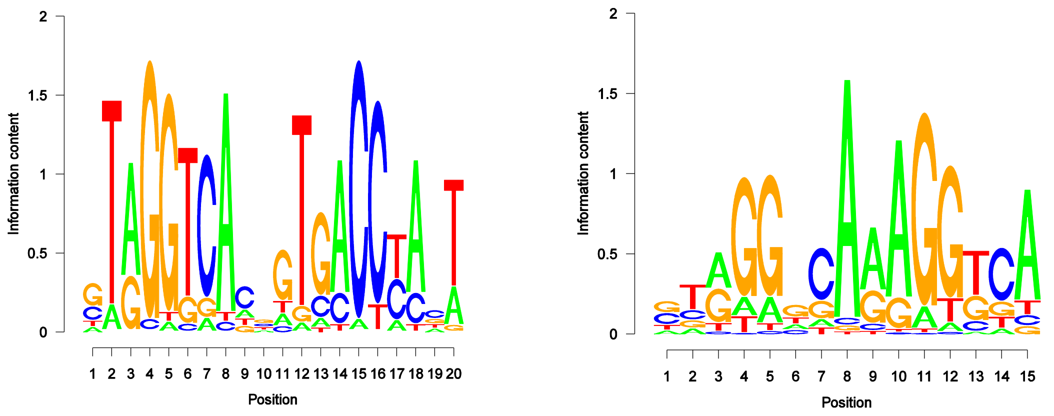

Fig. S7: The JASPAR motif logos. Left: PPARG; right: PPARG:RXR.

Table S16: The loadings and ranks of the genes downstream of the HIF-1 signaling pathway, other genes that stimulate angiogenesis, and genes in response to hypoxia, from the HFD end in the pancreatic first gene eigenvector.

	Symbol		Loading value	Ranks (from the HFD end)	Gene.Title
**HIF-1 signaling pathway and related**	*Car3*		0.13078	1	carbonic anhydrase 3
	Ang		0.05704	19	angiogenin, ribonuclease, RNase A family, 5
	Angptl4		0.02094	194	angiopoietin-like 4

**genes**					
	Efemp1		0.040482	49	epidermal growth factor-containing fibulin-like extracellular matrix protein 1
	Eps15		0.013884	439	epidermal growth factor receptor pathway substrate 15
	Egr1		0.04426	37	early growth response 1
	Flt1 (VEGFR-2)		0.00849	946	FMS-like tyrosine kinase 1
	Kdr (VEGFR-2)		0.00975	783	kinase insert domain protein receptor
	Tie-2 (Tek)		0.002875	2281	endothelial-specific receptor tyrosine kinase
	Timp4		0.05173	25	tissue inhibitor of metalloproteinase 4
	Timp2		0.026153	130	tissue inhibitor of metalloproteinase 2
	Timp3		0.021826	180	tissue inhibitor of metalloproteinase 3
	Serpine2		0.02367	157	serine (or cysteine) peptidase inhibitor, clade E, member 2
	Cav1		0.03701	62	caveolin 1, caveolae protein
	Hif1a		0.00518	1614	hypoxia inducible factor 1, alpha subunit
**Other angio-genesis stimulators**	*Fgf1*		0.027482	115	fibroblast growth factor 1
	Fgfrl1		0.014441	399	fibroblast growth factor receptor-like 1 / similar to fibroblast growth factor receptor 5 beta
	Tgfbi		0.029681	92	transforming growth factor, beta induced
	Tgfbr3		0.017585	272	transforming growth factor, beta receptor III
	Tgfbr2		0.016494	319	transforming growth factor, beta receptor II
	Ptgis		0.019271	233	prostaglandin I2 (prostacyclin) synthase
	Nrp1		0.014041	427	Neuropilin-1
**Hypoxia-related Genes (GO - Biological**	*Adipoq*		0.10891	3	adiponectin, C1Q and collagen domain containing
	Adipor2		0.012076	542	adiponectin receptor 2

**Process)**	*Cryab*		0.03753	61	crystallin, alpha B
	Cd38		0.02216	173	CD38 antigen
	Sod3		0.02138	187	superoxide dismutase 3, extracellular
	Atp1b1		0.02072	201	ATPase, Na+/K+ transporting, beta 1 polypeptide
	Bnip3		0.02043	204	BCL2/adenovirus E1B interacting protein 3
	Bcl2		0.0167	311	B-cell leukemia/lymphoma 2
	Vcam1		0.01641	323	vascular cell adhesion molecule 1
	Cxcl12		0.0157	351	chemokine (C-X-C motif) ligand 12
	Epas1		0.01544	365	endothelial PAS domain protein 1
	Plat		0.013838	441	plasminogen activator, tissue
	Pam		0.01368	446	peptidylglycine alpha-amidating monooxygenase
	Ddit4		0.01236	521	DNA-damage-inducible transcript 4
	Capn2		0.01082	666	calpain 2
	Rhoc		0.01065	678	ras homolog gene family, member C
	Fosl2		0.01053	686	fos-like antigen 2
	Sox4		0.0095	812	SRY-box containing gene 4
**Globin**	*Hp*		0.05889	15	haptoglobin
	Hba-a1		0.04066	48	hemoglobin alpha, adult chain 1 /hemoglobin alpha, adult chain 2
	Hbb-b1		0.02819	109	hemoglobin, beta adult major chain /hemoglobin, beta adult minor chain
**Iron**	*Trf*		0.06455	10	transferrin

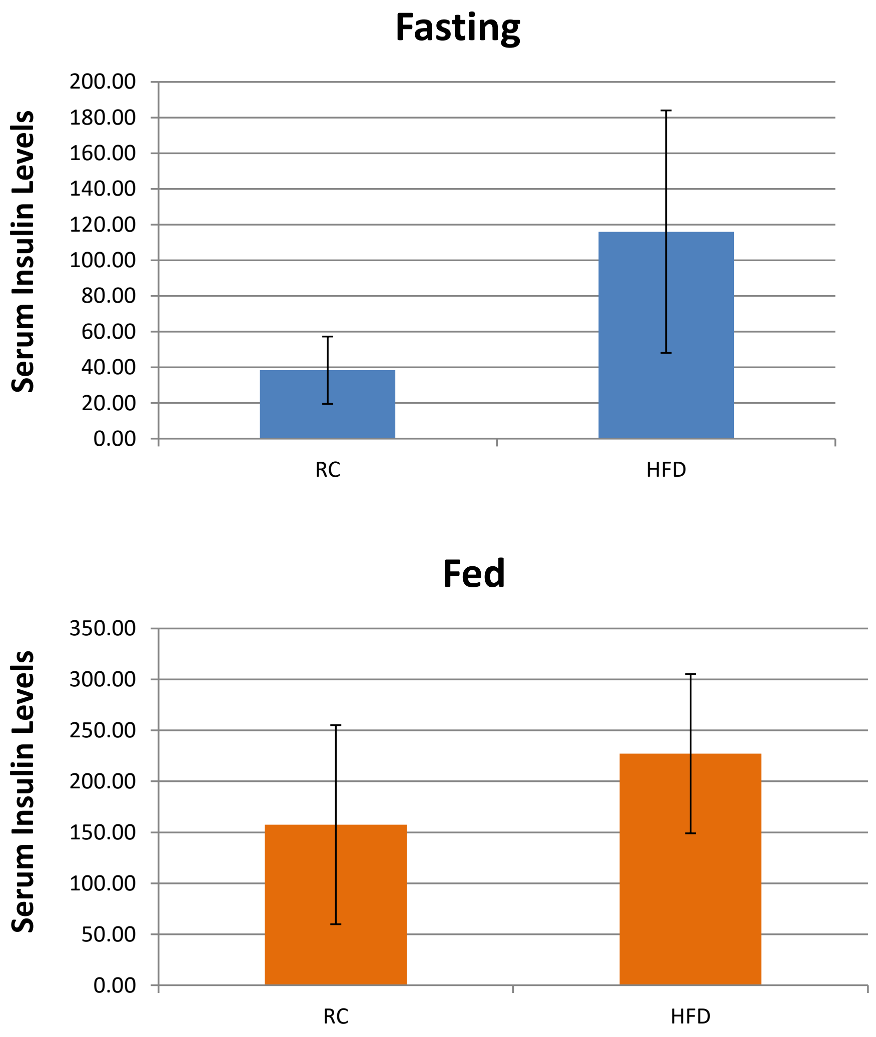

Fig. S8. Fasting and fed serum insulin levels of the mice fed with RC or with the HFD at the 18-week. The number of experimental mice was n = 3 (RC-fed mice) and n = 5 (HFD-fed mice).

References

1 Cluster and TreeView.

2 Cluster3.0.

3 Cheng, C. et al. Significant and systematic expression differentiation in long-lived yeast strains. PLoS One 2, e1095, doi:10.1371/journal.pone.0001095 (2007).

4 Wei, M. et al. Tor1/Sch9-regulated carbon source substitution is as effective as calorie restriction in life span extension. PLoS Genet 5, e1000467, doi:10.1371/journal.pgen.1000467 (2009).

5 Ge, H. et al. Comparative analyses of time-course gene expression profiles of the long-lived sch9Delta mutant. Nucleic Acids Res 38, 143-158, doi:10.1093/nar/gkp849 (2010).

6 Wang, L. et al. Induction of apoptosis through ER stress and TP53 in MCF-7 cells by the nanoparticle [Gd@C82(OH)22]n: A systems biology study. Methods 67, 394-406, doi:10.1016/j.ymeth.2014.01.007 (2014).

7 Erridge, C., Attina, T., Spickett, C. M. & Webb, D. J. A high-fat meal induces low-grade endotoxemia: evidence of a novel mechanism of postprandial inflammation. Am J Clin Nutr 86, 1286-1292 (2007).

8 Heilbronn, L. K. & Campbell, L. V. Adipose tissue macrophages, low grade inflammation and insulin resistance in human obesity. Curr Pharm Des 14, 1225-1230 (2008).

9 Xu, H. et al. Chronic inflammation in fat plays a crucial role in the development of obesity-related insulin resistance. J Clin Invest 112, 1821-1830, doi:10.1172/JCI19451 (2003).

10 Pai, J. T., Guryev, O., Brown, M. S. & Goldstein, J. L. Differential stimulation of cholesterol and unsaturated fatty acid biosynthesis in cells expressing individual nuclear sterol regulatory element-binding proteins. J Biol Chem 273, 26138-26148 (1998).

11 Kanehisa, M., Furumichi, M., Tanabe, M., Sato, Y. & Morishima, K. KEGG: new perspectives on genomes, pathways, diseases and drugs. Nucleic Acids Res 45, D353-D361, doi:10.1093/nar/gkw1092 (2017).

12 International Union of Biochemistry and Molecular Biology. Nomenclature Committee & Webb, E. C. Enzyme nomenclature 1992 : recommendations of the Nomenclature Committee of the International Union of Biochemistry and Molecular Biology on the nomenclature and classification of enzymes. (Academic Press, 1992).

13 Dahlquist, K. D., Salomonis, N., Vranizan, K., Lawlor, S. C. & Conklin, B. R. GenMAPP, a new tool for viewing and analyzing microarray data on biological pathways. Nat Genet 31, 19-20, doi:10.1038/ng0502-19 (2002).
